# Cell-Membrane-Coated and Cell-Penetrating Peptide-Conjugated Trimagnetic Nanoparticles for Targeted Magnetic Hyperthermia of Prostate Cancer Cells

**DOI:** 10.1021/acsami.3c07248

**Published:** 2023-06-13

**Authors:** Valentin Nica, Attilio Marino, Carlotta Pucci, Özlem Şen, Melis Emanet, Daniele De Pasquale, Alessio Carmignani, Andrea Petretto, Martina Bartolucci, Simone Lauciello, Rosaria Brescia, Francesco de Boni, Mirko Prato, Sergio Marras, Filippo Drago, Mohaned Hammad, Doris Segets, Gianni Ciofani

**Affiliations:** † Istituto Italiano di Tecnologia, Smart Bio-Interfaces, Viale Rinaldo Piaggio 34, 56025 Pontedera, Italy; ‡ Sant’Anna School of Advanced Studies, The Biorobotics Institute, Viale Rinaldo Piaggio 34, 56025 Pontedera, Italy; § IRCCS Istituto Giannina Gaslini, Core Facilities-Clinical Proteomics and Metabolomics, Via Gerolamo Gaslini 5, 16147 Genova, Italy; ∥ Istituto Italiano di Tecnologia, Electron Microscopy Facility, Via Morego 30, 16163 Genova, Italy; ⊥ Istituto Italiano di Tecnologia, Materials Characterization Facility, Via Morego 30, 16163 Genova, Italy; ¶ University of Duisburg-Essen, Particle Science and Technology - Institute for Combustion and Gas Dynamics (IVG-PST), Carl-Benz Strasse 199, 47057 Duisburg, Germany

**Keywords:** trimagnetic nanoparticles, cell membranes, cell-penetrating peptides, intracellular hyperthermia, prostate cancer

## Abstract

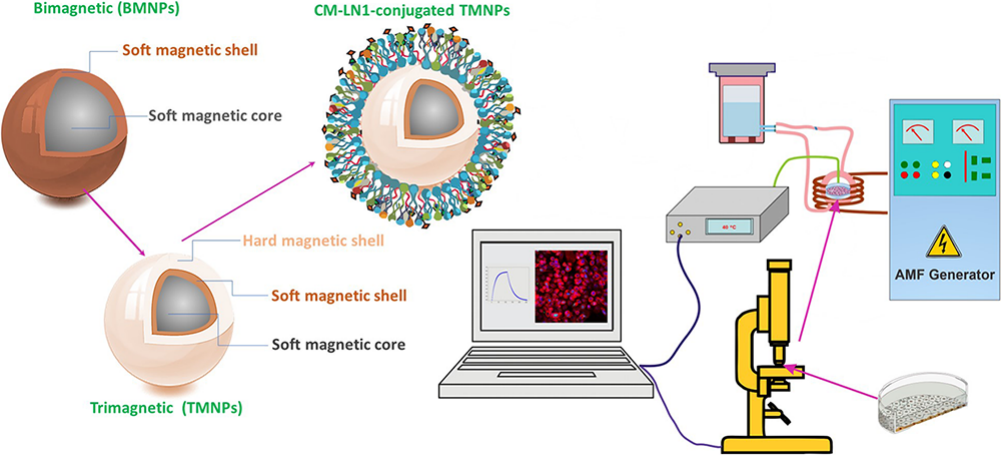

Prostate malignancy represents the second leading cause of cancer-specific death among the male population worldwide. Herein, enhanced intracellular magnetic fluid hyperthermia is applied *in vitro* to treat prostate cancer (PCa) cells with minimum invasiveness and toxicity and highly specific targeting. We designed and optimized novel shape-anisotropic magnetic core–shell–shell nanoparticles (i.e., trimagnetic nanoparticles - TMNPs) with significant magnetothermal conversion following an exchange coupling effect to an external alternating magnetic field (AMF). The functional properties of the best candidate in terms of heating efficiency (i.e., Fe_3_O_4_@Mn_0.5_Zn_0.5_Fe_2_O_4_@CoFe_2_O_4_) were exploited following surface decoration with PCa cell membranes (CM) and/or LN1 cell-penetrating peptide (CPP). We demonstrated that the combination of biomimetic dual CM-CPP targeting and AMF responsiveness significantly induces caspase 9-mediated apoptosis of PCa cells. Furthermore, a downregulation of the cell cycle progression markers and a decrease of the migration rate in surviving cells were observed in response to the TMNP-assisted magnetic hyperthermia, suggesting a reduction in cancer cell aggressiveness.

## 1 Introduction

Magnetic-fluid-mediated hyperthermia (MFH) is a promising technique for cancer treatment, proved by successful human clinical trials,^1^ and it is exploited in many types of tumors such as brain,^2^ prostate,^3^ breast,^4^ and pancreatic^5^ cancer. Prostate cancer (PCa) remains a very important key health concern, and it is the most common cancer in males worldwide. For instance, the malignant neoplasm of the prostate is a leading cause of death in men in Europe, accounting for 65 200 cases (2.9% of all male deaths) in 2018.^6^

Recent attainments of MFH in PCa are very promising; however, the method needs further development for clinical implementation, and^7^ the selection of the most suitable material and procedure aiming at efficient MFH is still a persistent matter of debate.^8^ Nevertheless, the development of high-efficiency nanoheaters showing a high specific absorption rate (SAR) value is a requisite for clinical application to overcome several limitations related to the nanoparticle quantity used in therapy and thus to their consequent toxicity.^9^ The toxicity of magnetic nanoparticles (MNPs) can indeed depend on many factors, such as size, shape, surface coating, composition, concentration, exposure time, route of administration, pharmacokinetics, and biodegradability.^10−12^ Some of the possible mechanisms of toxicity include oxidative stress, inflammation, genotoxicity, and immunotoxicity.^13,14^ These effects can vary depending on the type of cells or tissues that interact with the MNPs and their biological environment.^13^ Therefore, it is important to optimize the design of MNPs to minimize their toxicity and maximize their therapeutic effectiveness. Some of the strategies to achieve these purposes include choosing appropriate biocompatible materials and coatings, controlling the size and shape distribution, enhancing the stability and solubility, reducing the aggregation and sedimentation, regulating the mechanisms of local induction of heat in subcellular compartments, and improving the targeting and responsiveness of MNPs.^15,14^

There is rich scientific literature that outlines the improvement of SAR values by optimizing various chemical–physical parameters of the nanoparticles such as shape anisotropy, magnetic shell thickness, magnetic anisotropy, size, and cation distribution inside nanocrystals, or stability in polar and nonpolar solvents.^16,17^ It has been demonstrated that bimagnetic nanoparticles (BMNPs) have a SAR value of one order magnitude higher than single core nanoparticles.^18^ Other studies focused on magnetic systems in the ferromagnetic regime,^19^ whereas MNPs with superparamagnetic characteristics are more desirable for inductive hyperthermia. In addition, shape-anisotropic MNPs play a key role in increasing SAR for heat-mediated hyperthermia.^20^ We reported that superparamagnetic truncated-octahedron (Zn_*x*_Co_1–*x*_Fe_2_O_4_@MnFe_2_O_4_)^21^ and spherical (Zn_0.4_Co_0.6_Fe_2_O_4_@ Zn_0.4_Mn_0.6_Fe_2_O_4_)^22^ BMNPs are highly efficient in hyperthermia therapy. In another study, we synthesized polyhedron-shaped BMNPs (Zn_0.4_Co_0.6_Fe_2_O_4_@Zn_0.4_Mn_0.6_Fe_2_O_4_) which exhibit superparamagnetic properties and a 2-fold increase of SAR with respect to the core nanoparticles with the same volume and composition.^23^ However, there is a need to find new effective ways to demonstrate the therapeutic local effect of magnetic heat dissipation into the cell structure. For instance, intracellular hyperthermia has been claimed as an efficient strategy to increase apoptosis in glioblastoma cells.^24^

There are few papers reporting on the synthesis and characterization of trimagnetic nanoparticles (TMNPs). Isaac et al. reported on spherical multishell MnFe_2_O_4_@CoFe_2_O_4_@NiFe_2_O_4_ nanoparticles;^25^ these nanoparticles, having a mean size of 11 nm, behave as ferromagnetic structures with a mild saturation magnetization (*M*_S_ = 65 emu/g). Other researchers reported the fabrication of onion-like Fe_3_O_4_/MgO/CoFe_2_O_4_ core–shell–shell nanostructures with the magnetite mean core size of 22 ± 4 nm coated with an inner shell of magnesium oxide and an outer core of cobalt ferrite of thicknesses ≈1 and ≈2.5 nm, respectively. The magnetization measurements at the temperature *T* = 5 K revealed enhancement of the coercivity field, from *H*_C_ ≈ 48.3 kA/m for the Fe_3_O_4_/MgO to *H*_C_ ≈ 468.7 Oe for the Fe_3_O_4_/MgO/CoFe_2_O_4_ nanoparticles, attributed to the magnetic coupling between both ferrimagnetic phases.^26^ To the best of our knowledge, no investigations about SAR assessment of this kind of nanoparticles can be found in the literature, thus representing TMNPs as a viable alternative to conventional hyperthermia where high doses of therapeutic agents are required.^27−29^

Current research shows that the therapeutic efficacy of MFH is limited by poor stabilization of MNPs in aqueous environments and their bioavailability or by the lack of targeting specificity in tumors.^30^ By overcoming these limitations, the functionalization of MNPs with a biomimetic coating may significantly improve the feasibility of PCa therapy.^31,32^ Cell membranes exhibit unique functional components that can improve biocompatibility, cellular uptake, tumor-specific targeting, and site accumulation.^33^ Some authors reported an enhancement of both magnetic resonance imaging (MRI) signal and MCF-7 human breast tumor photothermal therapy efficiency following the functionalization of Fe_3_O_4_ nanoparticles with red blood cell membrane-derived vesicles.^34^ In another example, a myeloid-derived suppressor cell membrane was coated onto spherical Fe_3_O_4_ nanoparticles to target C–C or C–X–C chemokine receptors.^35^ Cell-penetrating peptides (CPPs) are instead known to facilitate targeting with limited toxicity^27^ and noninvasiveness:^36^ very recently, a novel tissue-specific CPP displayed high targeting affinity for LNCaP prostate cancer cells *in vivo*.^37^ LN1 showed cell-specific selectivity to tumor tissue, avoiding adverse reactions on healthy liver and kidney cells.

In the current study, we designed and developed novel magnetically core–shell–shell nanoparticles (namely trimagnetic nanoparticles, TMNPs) with enhanced SAR for intracellular hyperthermia against PCa cells. We demonstrated that the functionalization of lipid-PEGylated TMNPs with both cell membrane (CM) and LN1 CPP induces a strong selectivity for PCa cells. We therefore investigated the therapeutic effects of TMNPs-mediated hyperthermia, evaluating cellular death, migration rate, and molecular mechanisms at the base of the observed phenomena.

## 2 Experimental Section

### 2.1 Synthesis of Bimagnetic (BMNPs) and Trimagnetic Nanoparticles (TMNPs)

Trimagnetic nanoparticles (TMNPs) with magnetic core–shell–shell architecture have been synthesized in a two-step procedure by thermal decomposition of metallic complexes and a subsequent one-step seed-mediated growth route. In the first step, highly monodispersed core–shell bimagnetic nanoparticles (BMNPs) have been obtained in a slightly modified one-pot synthesis.^38^ For a magnetically soft–soft system (Fe_3_O_4_@Mn_0.5_Zn_0.5_Fe_2_O_4_, further noted as SS), 1.334 mmol of iron(III) acetylacetonate (Fe(acac)_3_, 99%, Aldrich), 0.333 mmol of manganese(II) acetylacetonate (Mn(acac)_2_, 95%, Aldrich), and 0.333 mmol of zinc acetylacetonate (Zn(acac)_2_, 98%, Aldrich), were added in a solution of 6 mmol of 1,2-hexadecandiol (98%, Sigma-Aldrich), 6 mmol of oleic acid (90%, Aldrich), and 6 mmol of oleylamine (98%, Sigma-Aldrich) in 20 mL of benzyl ether (98%, Sigma-Aldrich). The mixture was mechanically stirred under nitrogen flow and heated at 80 °C for 240 min under vacuum. Then the reaction mixture was heated at 200 °C for 2 h and to reflux for 1 h with a heating rate of 12 °C/min. The particles were washed three times with ethanol (99%, Sigma-Aldrich) and hexane (99.9%, Carlo Erba) and separated by centrifugation (10 min, 10000*g*, 4 °C, Thermo Scientific Sorvall Lynx 4000). In the second step, 80 mg of BMNPs dispersed in chloroform (99.8%, Sigma-Aldrich) was added to a reaction system containing 0.250 mmol of Fe(acac)_3_, 0.125 mmol of cobalt(II) acetylacetonate (Co(acac)_2_, 99.9%, Aldrich), 3 mmol of oleic acid (OA), and 3 mmol of oleylamine (OAm). To grow the second magnetic shell (i.e., CoFe_2_O_4_) on the surface of the BMNPs, we used similar experimental conditions described in the first step but with a reaction time of 30 min under reflux. After three cleaning and centrifugation steps, the Fe_3_O_4_@Mn_0.5_Zn_0.5_Fe_2_O_4_@CoFe_2_O_4_ magnetic soft–soft–hard TMNPs (SSH) were dispersed in chloroform.

Analogously, by changing the molar ratio of metallic compounds for each sample and its core–shell–shell architecture, we synthesized another type of BMNPs (Fe_3_O_4_@Co_0.5_Zn_0.5_Fe_2_O_4_, indicated as SH) and of TMNPs with magnetic soft–hard–soft architecture (Fe_3_O_4_@Co_0.5_Zn_0.5_Fe_2_O_4_@MnFe_2_O_4_, indicated as SHS), respectively. The experimental conditions (i.e., synthesis time, reflux time, nitrogen flux, cleaning steps) for these samples were similar to those described for SS and SSH, respectively.

### 2.2 Water-Phase Transfer of SH, SHS, SS, and SSH MNPs

The thermal decomposition procedure provides magnetic nanoparticles dispersed in organic solvents unsuitable for most biological purposes. In a typical synthesis route, the hydrophobic SSH MNPs were thus transferred to an aqueous medium by exploiting a dual solvent exchange protocol.^39^ The method provides high water dispersibility through phospholipid–PEG coating, with desirable reactive groups on the MNP surface. To facilitate the ligand exchange, 3.2 mL of SSH MNPs dispersed in chloroform (16 mg Fe/mL) was added to a mixture containing a 3:1 weight ratio of 1,2-distearoyl-*sn*-glycero-3-phosphoethanolamine-*N*-[amino(polyethylene glycol)-5000] (ammonium salt) (DSPE-mPEG, 5000 Da, Nanocs, Inc.)/1,2-distearoyl-*sn*-glycero-3-phosphoethanolamine-*N*-[maleimide(polyethylene glycol)-5000] (ammonium salt) (DSPE-PEG maleimide, 5000 Da, Avanti Polar Lipids). The amount of phospholipid–polymer dissolved in chloroform was calculated as a 1:2 weight ratio between polymers and iron content. An amount of 60 mL of dimethyl sulfoxide (DMSO, 99.9%, Thermo Scientific) was dripped gradually during the reaction, and the resulting mixture was incubated on a shaker at 25 °C for 1 h. The chloroform was removed overnight under vacuum; afterward, 30 mL of Milli-Q water (Millipore) was added to form a colloidal dispersion. The excess amphiphilic micelles and DMSO have been removed by three centrifugation steps (20 min, 10000*g*, 10 °C). The coated SSH MNPs were finally redispersed in 4 mL of Milli-Q water and denoted as L-SSH.

Using a similar coating procedure, SHS, SS, and SH MNPs have been transferred into an aqueous medium and further denoted as L-SHS, L-SS, and L-SH MNPs, respectively.

### 2.3 Functionalization of L-SSH MNPs with a Cell Membrane and Cell-Penetrating Peptide

L-SSH MNPs have been functionalized with CM or with LN1 CPP similarly as previously reported by our group.^40,41^ A double functionalization with CM/LN1 has been considered as well.

#### 2.3.1 Cell Membrane Extraction

PCa-derived cell membranes have been obtained via hypotonic cell lysis, mechanical membrane disruption by high-pressure homogenization, and differential centrifugation. The PC-3 (ATCC CRL-1435) caucasian prostate adenocarcinoma cell line has been used for cell membrane extraction. PC-3 cells were cultured using the Gibco Roswell Park Memorial Institute 1640 medium (RPMI, Gibco, ThermoFisher) supplemented with 1% penicillin–streptomycin (P/S), 1% l-glutamine, and 10% fetal bovine serum (FBS, Sigma-Aldrich). PC-3 cells (10^4^ cells/cm^2^) were seeded in 10 cm diameter Petri dishes treated for cell culture (Corning). When the cells grew at about 90% of confluence, they were washed twice with Dulbecco’s phosphate buffer saline solution (DPBS, Euroclone) and detached in 4 mL of DPBS per dish. After a centrifugation step (660*g* for 5 min), pellets were resuspended in cold (4 °C) Milli-Q water, and cells were disrupted with a high-pressure homogenizer (20 psi). Samples were centrifuged at 10000*g* for 10 min at 4 °C, and the supernatant containing CM was collected and further centrifuged at 37000*g* for 60 min at 4 °C. The obtained pellet was finally resuspended in 1 mL of Milli-Q water.

#### 2.3.2 L-SSH MNP Coating with a Cell Membrane

An amount of 1 mL of CM extract derived from 5 × 10^6^ cells was used to coat 2 mg of L-SSH MNPs dispersed in Milli-Q water (2 mg/mL). The membrane coating was attained through ultrasonic high power (25 W) treatment (20 kHz, Fisherbrand Q125 Sonicator, FisherScientific) with intermittent pulse timing mode (2 s pulse, 30 min) in an ice bath. The sample was thereafter washed three times with deionized water and susequently collected by centrifugation (16000*g*, 90 min, 4 °C). The pellet was eventually disersed in 1 mL of Milli-Q water and the final product indicated as CM-L-SSH.

#### 2.3.3 Functionalization of L-SSH MNPs with an LN1 Cell-Penetrating Peptide

The L-SSH MNP surface was modified with a CPP (LN1, 95%, CTGTPARQC sequence, ProteoGenixSAS) using maleimide–thiol Michael addition click reaction involving the DSPE-PEG maleimide terminal group and the cysteine end group of the peptide sequence. An amount of 50 μL of LN1 peptide aqueous solution (1 mg/mL) was mixed with 1 mL of L-SSH dispersion (4 mg/mL) and diluted with PBS to a neutral pH for site-selective reaction of cysteine. The product was maintained in an ice bath and gently shaken for 4 h in the dark. Then, the sample was washed through centrifugation (16000*g*, 90 min, 4 °C) three times and eventually dispersed in 1 mL of Milli-Q water. The sample is named LN1-L-SSH.

#### 2.3.4 Functionalization of L-SSH MNPs with a Cell Membrane and LN1 Cell-Penetrating Peptide

**Figure 1:**
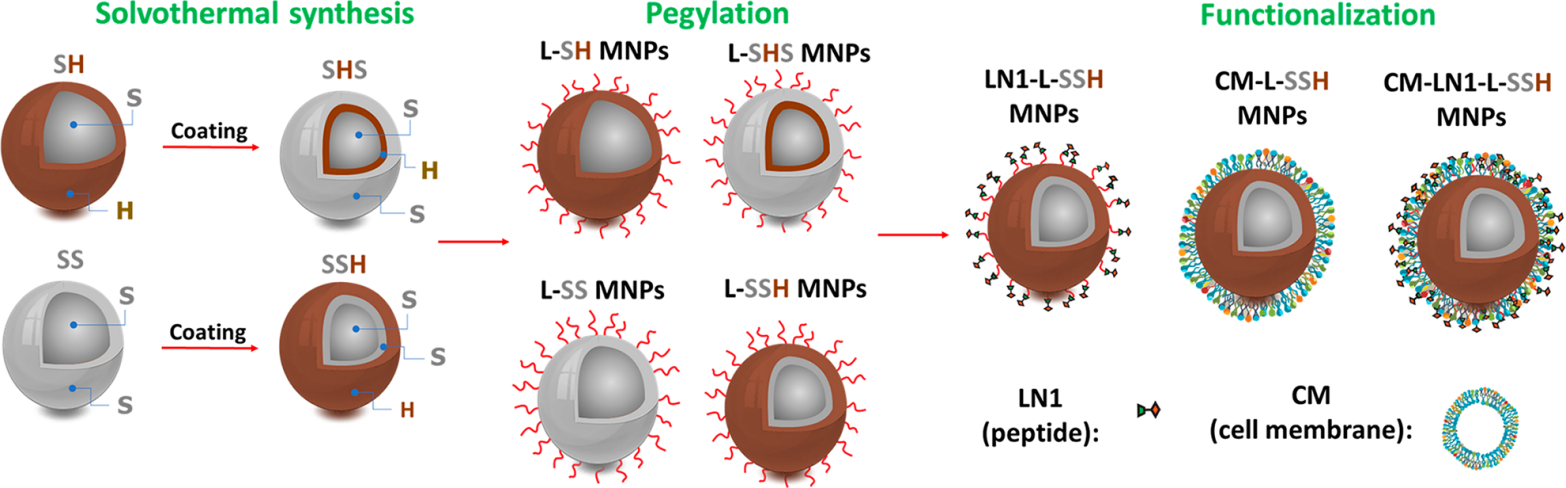
Schematic illustration of coating and functionalization procedures of BMNPs and TMNPs: Fe_3_O_4_@Co_0.5_Zn_0.5_Fe_2_O_4_, soft–hard (SH); Fe_3_O_4_@Co_0.5_Zn_0.5_Fe_2_O_4_@MnFe_2_O_4_, soft–hard–soft (SHS); Fe_3_O_4_@Mn_0.5_Zn_0.5_Fe_2_O_4_, soft–soft (SS); (c) Fe_3_O_4_@Mn_0.5_Zn_0.5_Fe_2_O_4_@CoFe_2_O_4_, soft–soft–hard (SSH).

An amount of 1 mL of CM and LN1 (10:1 w/w) aqueous solution (pH ∼ 7) was added into 1 mL of L-SSH MNP dispersion (4 mg/mL) under high power intermittent sonication (25 W, 2 s/pulse, 30 min) at 4 °C. Then, the mixture was placed in an ice bath on a shaking plate for 4 h in the dark. The sample followed the same procedure of cleaning and centrifugation as previously described, and finally, it was dispersed in 1 mL of Milli-Q water. The sample is named CM-LN1-L-SSH. A representative illustration of CM-LN1-L-SSH MNP preparation and their application for *in vitro* prostate cancer treatment is presented in Figure S1 (Supporting Information), while the schematic representation of MNPs’ coating and their functionalization is given in Figure 1.

#### 2.3.5 Fluorescence Staining

For confocal microscopy imaging, L-SSH, CM-L-SSH, LN1-L-SSH, and CM-LN1-L-SSH MNPs were stained with a fluorescent lipophilic dye (Vybrant DiO, ThermoFisher) by incubating 1 mg of nanoparticles with 10 μL of dye (2 h, 37 °C). The samples were washed three times by centrifugation (16 000*g*, 90 min, 4 °C) before use.

### 2.4 Physical–Chemical Characterization

Bright-field (BF) transmission electron microscopy (TEM) micrographs were obtained with a JEOL JEM-1011 (JEOL Ltd.) operated at 100 kV. The samples were gently sonicated (5 min), and a drop of the suspension was dried on a carbon-coated Cu grid (150 mesh). The mean diameter of pristine MNPs has been obtained by Gaussian fitting of the size distribution curve using the ImageJ software package^42^ (version 1.53s). For CM-L-SSH and CM-LN1-L-SSH MNPs, the samples were gently sonicated (5 min), and a drop of the suspension was dried on a carbon-coated Cu grid (150 mesh). To highlight the lipid coating, the samples were negatively stained with a solution of 1% uranyl acetate in water for 30 s, and then TEM grids were dried in air. Energy-filtered TEM (EFTEM) imaging was carried out on an image-Cs-corrected JEOL JEM-2200FS transmission electron microscope (TEM) equipped with an in-column imaging filter (Ω-type), operated at 200 kV. The EFTEM elemental mapping was obtained by the three-window method at the L_23_ edges of Mn (onset *E* = 640 eV, slit width Δ*E* = 30 eV), Co (*E* = 779 eV, Δ*E* = 16 eV), Fe (*E* = 708 eV, Δ*E* = 16 eV), and Zn (*E* = 1020 eV, Δ*E* = 60 eV): width and position of the energy-selecting slit were optimized to minimize overlaps from neighboring ionization edges.

Scanning electron microscopy (SEM) has been used to assess the morphology of the samples and, in particular, the shape of the nanoparticles. Images were acquired by using a Helios NanoLab 600 DualBeam FIB/SEM (FEI) instrument (voltage of 2 kV, beam current of 0.04 nA, and dwell time set to 8 μs). The samples were prepared by dropping the nanoparticle dispersion directly on a carbon-coated holder and drying at room temperature under a flow of argon gas.

X-ray photoelectron spectroscopy (XPS) measurements were carried out through a Kratos Axis Ultra^DLD^ spectrometer (Kratos Analytical Ltd.) with a monochromated Al K_α_ X-ray source (*hν* = 1486.6 eV) operating at 20 mA and 15 kV. The specimens were prepared by pressing a low amount of fine-grounded sample onto a high-purity indium pellet (99.9%, Sigma-Aldrich). The wide scans were collected over an analysis area of 300 × 700 μm^2^ at a photoelectron pass energy of 160 eV and energy step of 1 eV, while high-resolution spectra were collected at a photoelectron pass energy of 20 eV and an energy step of 0.1 eV. Differential electrical charging effects on the surface of the sample were neutralized during the measurements of all specimens. The spectra have been referenced to the adventitious carbon 1s peak at 284.8 eV. The spectra were analyzed with the CasaXPS software (Casa Software Ltd., version 2.3.24),^43^ and the residual background was eliminated by the Shirley method.

Inductively coupled plasma optical emission spectroscopy (ICP-OES, iCAP-7600 DUO, ThermoFisher) has been performed to evaluate the elemental composition of pristine BMNPs and TMNPs (SH, SS, SHS, and SSH MNPs). The samples were digested with 800 μL of nitric acid (HNO_3_ 60% v/v) and 200 μL of hydrogen peroxide (H_2_O_2_ 30%) under sonication (65 °C for 2 h). Before measurements, 100 μL of each sample was diluted in a 10 mL vial by adding deionized water. All measurements have been carried out using a plasma power of 1150 W, a nebulizer gas flow of 0.5 L/min, a cooling flow of 12 L/min, and an auxiliary flow of 0.5 L/min. The amount of Co, Mn, Zn, and Fe was determined. All chemical analyses performed by ICP-OES were affected by a systematic error of 5%.

X-ray diffraction (XRD) analysis was performed on a third-generation Empyrean X-ray diffractometer (Malvern Panalytical), equipped with a MoK_α_ (λ = 0.71 Å) ceramic X-ray tube (60 kV, 40 mA) and a GaliPIX^3D^ solid-state pixel detector. The diffraction patterns were collected in environmental atmosphere at room temperature within the range 2θ = 20–100°. The samples gripped on mylar foil substrate were mounted in a reflection-transmission spinner sample stage (rotation speed = 1 rps) and analyzed in transmission geometry. The mean crystallite size (*d*_XRD_) and the lattice constant (*a*) were calculated from the diffractograms through the Pawley method (PANalytical Software version 3.0, PANalytical B.V.),^44^ which uses whole-pattern profile fitting of the XRD pattern without a structural model.

The size distribution and *Z*-potential analyses of L-SSH, CM-L-SSH, LN1-L-SSH, and CM-LN1-L-SSH MNPs were performed by using a Zetasizer Nano ZS90 (Malvern Instruments, Ltd.). The measurements have been performed on 100 μg/mL nanoparticle dispersions in Milli-Q water at 37 °C with a scattering angle of 90°. The mean hydrodynamic diameter and polydispersity index (PDI) parameters were determined from the fitting (cumulant analysis) of the measured intensity by autocorrelation function. The *Z*-potential was determined by adjusting the conductivity of the sample in the range 10–100 μS/cm. The stability of L-SSH and CM-LN1-L-SSH MNPs, respectively, in Milli-Q water, DMEM, and PBS was assessed by evaluating the trend of the hydrodynamic diameter over time (up to 1 month). Under the same experimental conditions, the stability study of CM-LN1-L-SSH MNPs has been performed in a serum-containing physiological simulated medium, i.e., 90% DMEM + 10% FBS. Each measurement has been performed in triplicate, on three different sample preparations.

Thermogravimetric analysis (TGA) was carried out by using a TGA Q50 device (TA Instruments). L-SSH, CM-L-SSH, LN1-L-SSH, and CM-LN1-L-SSH nanoparticles were analyzed within the temperature range of 30–600 °C under a N_2_ flow (50 mL/min).

The bicinchoninic acid assay (BCA) quantification method is generally used in the literature to quantify peptide/protein functionalization in inorganic and hybrid membrane-derived nanoparticles with high sensitivity.^45,46^ It has been thus exploited (Pierce Protein Assay Kit, Thermo Scientific) to quantify the protein amount on CM-, LN1-, and CM/LN1-functionalized L-SSH MNPs. A standard protocol provided by the manufacturer was followed. Briefly, 25 μL of 4 mg/mL dispersion of each kind of nanoparticle (CM-L-SSH, LN1-L-SSH, CM-LN1-L-SSH MNPs) was mixed with 200 μL of standard assay solution on the microplate. The specimens were incubated at 37 °C on a shaker (60 rpm) for 30 min and subsequently centrifuged (10 min at 14000*g*). The supernatant of each sample was collected, and its absorbance (λ = 560 nm) was assessed with a UV–vis plate reader (Victor X3, PerkinElmer). For the LN1 quantification, the absorbance of L-SSH MNPs was subtracted. The protein amount was calculated using a calibration curve (0–1000 μg/mL albumin standard) and expressed for 1 mg/mL of solution.

The magnetic characterization has been carried out using a Physical Property Measurement System (Quantum Design PPMS DynaCool, Quantum Design). All experiments were performed at room temperature (300 K) over a maximum applied magnetic field of *B* = ±9 T.

Magnetic inductive measurements were performed using a MagneTherm instrument (NanoTherics) running at an applied external alternating magnetic field (AMF) of *B* = 20 mT (*H* = 15.9 kA/m) and a frequency *f* = 97.5 kHz. The specific absorption rate (SAR) was determined for the quantification of the heat dissipation rate of L-SHS, L-SH, L-SSH, and L-SS MNPs dispersed in an aqueous medium. A vial containing 100 μL of MNP ferrofluid sample with a concentration of 5 mg/mL was wrapped in styrofoam to reduce heat losses and placed at the center of a 17-turn water-cooled magnetic induction coil. The specimens were thermostated at an initial temperature of *T*_0_ = 298.15 K and then exposed to the AMF for 7 min. The temperature change over time was recorded using a fiber optic temperature sensor (OSENSA Innovations Corp.). The heating efficiency has been calculated using the corrected-slope method in agreement with eq 1:^47^
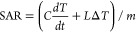
1where *C* is the specific heat capacity of water (4.182 J/kg K), *dT*/*dt* is the initial slope of the time vs temperature curve, *L* represents the linear loss parameter, Δ*T* is the average temperature difference between the sample and baseline, and *m* represents the mass of the nanoparticles.

At a low-frequency regime and low field strength, the intrinsic loss parameter (ILP) has been considered^36^ to compare the heating efficiency in various experimental conditions independently from the applied AMF characteristics (eq 2):
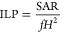
2where *H* represents the intensity of the applied field and *f* is the excitation frequency.

### 2.5 Biological Characterization

#### 2.5.1 Cell Viability Assays

The cytotoxicity tests were performed on the PC-3 (ATCC CRL-1435) Caucasian prostate adenocarcinoma cell line and on human primary normal prostate epithelial cells (ATCC-PCS-440-010). PC-3 cells were cultured in T175 flasks using the RPMI 1640 medium (Sigma) supplemented with 10% fetal bovine serum (FBS), 100 IU/mL of penicillin (Gibco), 100 μg/mL of streptomycin (Gibco), and 2 mM l-glutamine (Gibco). Human primary normal prostate epithelial cells were cultured with Prostate Epithelial Cell Basal Medium (ATCC) supplemented with Prostate Epithelial Cell Growth Kit (ATCC) as described by the manufacturer protocol. Cell viability was assessed using the WST-1 assay (Roche). PC-3 and human primary normal prostate epithelial cells were seeded in 24-well plates at the density of 15 × 10^3^ cells/cm^2^, and cell viability was evaluated upon 72 h treatment with L-SSH MNPs at increasing concentrations (0, 30, 85, 250, 500, and 700 μg/mL). Cultures were incubated with 300 μL of phenol red-free complete medium with the WST-1 reagent (1:20 dilution) for 30 min at 37 °C; thereafter, the absorbance of the supernatant was measured at 450 nm using a Victor X3 (PerkinElmer) UV–Vis plate reader. The absorbance values were expressed as % with respect to the control.

#### 2.5.2 Targeting Efficiency of L-SSH, CM-L-SSH, LN1-L-SSH, and CM-LN1-L-SSH MNPs

The cellular uptake was assessed by confocal laser scanning microscopy (CLSM) imaging (C 2s, Nikon). In a typical route, the PC-3 cells were seeded on WillCo glass dishes (15 × 10^3^ cells/cm^2^), incubated at 37 °C, and treated for 72 h with 250 μg/mL of dye-labeled L-SSH MNPs. Then, the sample was washed twice with PBS, fixed with 4% paraformaldehyde (PFA, Sigma-Aldrich) for 30 min at 4 °C, and finally stained with tetramethylrhodamine (TRITC)-phalloidin (100 μM, Millipore) and Hoechst 33342 (1 μg/mL, Invitrogen). The same procedure was performed for CM-L-SSH, LN1-L-SSH, and CM-LN1-L-SSH MNPs. Due to the superior targeting ability of CM-LN1-L-SSH MNPs to target PC-3 cells, the following experiments on magnetothermal stimulation have been carried out using this sample.

ICP-OES has been performed to evaluate the intracellular uptake of functionalized MNPs (L-SSH, CM-L-SSH, LN1-L-SSH, and CM-LN1-L-SSH MNPs). For the quantitative analysis of functionalized MNP internalization, PC-3 cells were seeded in T75 flasks (15 × 10^3^ cells/cm^2^) for 72 h, washed twice with PBS, detached with 0.05% trypsin-EDTA, and centrifuged. Then, the samples were digested and measured in a similar manner used for the ICP-OES analysis of pristine MNPs (SH, SHS, SS, and SSH MNPs). Finally, data were normalized for the nonfunctionalized controls (L-SSH MNPs).

#### 2.5.3 Magnetothermal Stimulation

The effects of CM-LN1-L-SSH MNP-mediated hyperthermia on cell viability, proliferation, apoptosis, and necrosis have been investigated under magnetothermal stimulation. PC-3 cells were seeded (15 × 10^3^ cells/cm^2^) and treated with 250 μg/mL of CM-LN1-L-SSH MNPs in HEPES-supplemented complete medium to keep the pH value stable during the AMF stimulation. Nonstimulated cultures were kept outside of the incubator for 100 min in HEPES-supplemented complete medium. Similarly as previously performed,^18,41^ the cultures underwent alternating magnetic field (AMF) stimulations (*f* = 97.5 kHz, *B* = 20 mT; 100 min/day stimulation over 3 days) after 24 h of magnetic nanoparticle incubation. CM-LN1-L-SSH MNP-treated AMF-stimulated cells (“CM-LN1-L-SSH MNPs + AMF”) were compared to nontreated nonstimulated control cells (“L-SSH MNPs”) as well as to samples stimulated with AMF but not treated with nanoparticles (“L-SSH MNPs + AMF”) and cells incubated with 250 μg/mL of CM-LN1-L-SSH MNPs but not stimulated with AMF (“CM-LN1-L-SSH MNPs”). WST-1 cell viability assay for the evaluation of CM-LN1-L-SSH MNP effects after the hyperthermia treatment has been carried out as described above.

The intraparticle temperature was recorded by CLSM using the DiI fluorescent dye similarly as previously described.^48^ Briefly, 0.5 mL of CM-LN1-L-SSH MNP (6 mg/mL) were stained with 20 μL of DiI staining solution (Vybrant Multicolor Cell-Labeling Kit, Invitrogen, ThermoFisher) for 30 min. Subsequently, the nanoparticles were washed 3 times by centrifugation and discarding the supernatant. PC-3 cells were seeded at 15 × 10^3^ cells/cm^2^ density and then treated with 250 μg/mL of DiI-stained CM-LN1-L-SSH MNPs. At 24 h of incubation, the cultures underwent alternating magnetic field (AMF) stimulations (by using the live cell coil of the MagneTherm equipment, NanoTherics; *f* = 97.5 kHz, *B* = 20 mT). During stimulation, time-lapse CLSM imaging was carried out with a confocal fluorescence microscope (CS2, Nikon) using the perfect focus configuration. To avoid objective heating during AMF, the microscope revolver was lowered by using the escape modality at the end of each acquisition (NIS-Elements software). The objective was automatically positioned in the perfect focus position just before carrying out the image acquisition. The fluorescence intensities of the regions of interest were then converted to temperatures by using the Δ*F*/*F*_0_ = −0.0224·Δ*T* linear function.^41^

#### 2.5.4 Immunofluorescence

Immunofluorescence was performed on “Control”, “Control + AMF”, “CM-LN1-L-SSH MNPs”, and “CM-LN1-L-SSH MNPs + AMF” experimental classes.

For the analysis of the *K*_i_-67 proliferation marker, the following procedure has been performed: after AMF stimulation, the cultures were washed three times with PBS, fixed with 4% PFA for 30 min at 4 °C, and then processed for immunofluorescence. Specifically, cell membranes were permeabilized by using Triton X-100 solution (0.1% v/v dilution, Sigma-Aldrich) in PBS for 30 min at room temperature. A blocking step was carried out by incubating samples with 10% goat serum in PBS for 45 min at room temperature. A 90 min treatment with a primary rabbit IgG anti-*K*_i_-67 antibody (1:150 dilution in PBS, Millipore) was performed at 37 °C. After subsequent washing steps (four times, 5 min each, by using PBS supplemented with 10% goat serum), cells were incubated with a 10% goat serum solution in PBS supplemented with a goat Alexa Fluor 488-IgG antirabbit secondary antibody (1:150 dilution, Invitrogen), Hoechst 33342 (1 μg/mL) for nuclei staining, and TRITC-phalloidin (120 μM) for F-acting staining. CLSM was carried out with a C 2s system (Nikon) using the autofocus mode and the same acquisition parameters for all samples. The total number of cell nuclei and of *K*_i_-67^+^ nuclei were counted in semiautomatic mode with the NIS-Elements software (Nikon) using the same signal thresholding parameters for the different experimental classes.

The heat shock protein 70 (hsp70) is a chaperone protein overexpressed in cancer cells exposed to magnetic fluid hyperthermia.^70^ For the analysis of hsp70 expression, the same procedures described above for *K*_i_-67 immunofluorescence have been followed, but an anti-hsp70 rabbit polyclonal IgG primary antibody (1:200 dilution, Genetex) and an Atto 488 goat antirabbit secondary antibody (1:300 dilution, Sigma-Aldrich) have been used. CLSM was carried out with a C 2s system (Nikon) using the autofocus mode and the same acquisition parameters. Finally, the signal intensity of hsp70 was measured using the NIS-Elements software (Nikon).

#### 2.5.5 Flow Cytometry

Flow cytometry analysis was carried out on “Control”, “Control + AMF”, “CM-LN1-L-SSH MNPs”, and “CM-LN1-L-SSH MNPs + AMF” experimental classes.

For the analysis of apoptosis and necrosis, cells after magnetothermal treatment were washed twice with PBS without Ca^2+^ and Mg^2+^ and detached with 0.5% trypsin for 6 min at 37 °C. Subsequently, the cells were centrifuged (7 min at 350 rpm), and the pellet was resuspended in annexin V binding buffer (1 ×) supplemented with 1.2 μg/mL of propidium iodide (PI) and 3 μM of annexin V-FITC (annexin V-FITC Apoptosis Staining/Detection Kit, Abcam) and left in the incubator at 37 °C for 15 min. The PI was used to stain necrotic cells, while the annexin V-FITC allowed the identification of apoptotic phenomena: the positivity to the annexin V-FITC marker is characteristic of the early apoptotic cells, while the double positivity to PI and annexin V-FITC is associated with late apoptosis. The percentages of viable (double negativity to both markers), necrotic, early apoptotic, and late apoptotic cells were analyzed by flow cytometry (FITC with λ_ex_ = 488 nm and λ_em_ = 505–545 nm, ECD-A with λ_ex_ = 488 nm and λ_em_ = 600–630 nm; Beckman Coulter CytoFLEX) using the CytoFLEX software.

Caspase-9 represents an initiator caspase, a cysteine-aspartic protease critical in triggering apoptotic signaling. Caspase-9 is active once integrated into the apoptosome, a large multimeric complex. The phosphorylation of caspase-9 inhibits the formation of the apoptosome.^49^ The detection of the activated form of caspase-9 was performed by using the CaspGLOW fluorescein active caspase-9 staining kit (Biovision), following the manufacturer’s procedures, on “Control”, “Control + AMF”, “CM-LN1-L-SSH MNPs”, and “CM-LN1-L-SSH MNPs + AMF” experimental classes, 60 min after a single stimulation session with AMF. The fluorescein isothiocyanate (FITC)-conjugated LEHD-FMK, a specific ligand of caspase-9 activated form, was utilized for detection. Briefly, cells were incubated for 1 h with 150 μL of phenol red-free DMEM supplemented with 1 μL of the FITC-LEHD-FMK caspase-9 fluorescent dye. Cells were subsequently washed twice with the appropriate wash buffer provided by the manufacturer. The cells were detached by using 0.05% trypsin/EDTA, centrifuged, and finally resuspended in PBS for flow cytometer analysis (FITC with λ_ex_ = 488 nm and λ_em_ = 505–545 nm). Fluorescence distributions were analyzed by using the CytoFLEX software. To identify the population of caspase-9 positive cells, the background fluorescence of nonstained cells was measured. Specifically, the highest fluorescence value of the nonstained controls was used to define the caspase-9 signal threshold: the population having a fluorescence intensity higher than the threshold was considered positive for the caspase-9 activation.

#### 2.5.6 Migration Assay

The migration rate of PC-3 cells in four experimental classes (“Control”, “Control + AMF”, “CM-LN1-L-SSH MNPs”, and “CM-LN1-L-SSH MNPs + AMF”) was assessed using a scratch assay. The cells were seeded in 2-well culture insert systems (80209, Ibidi) at a seeding density of 1 × 10^5^ cells/cm^2^ and incubated for 24 h. Then, they were treated with the plain medium as a control or with 250 μg/mL of CM-LN1-L-SSH MNPs for 24 h. After incubation, the culture insert was removed, and the cells were rinsed with PBS. The cultures were stained with 1 μM calcein (C3099, Invitrogen) for 15 min at 37 °C and then stimulated for 100 min with AMF as previously described. The gap between the cells (500 ± 100 μm) was imaged before starting AMF stimulation (*t* = 0 h) and after the stimulation (*t* = 24 h) by using a fluorescence microscope (Eclipse Ti, Nikon). The images were analyzed using ImageJ software^43^ (version 1.53s) with the “Wound Healing” plug-in.

#### 2.5.7 Proteomics

Samples of “Control”, “Control + AMF”, “CM-LN1-L-SSH MNPs”, and “CM-LN1-L-SSH MNPs + AMF” experimental classes were lysed, reduced, and alkylated in 50 μL of iST-LYSE buffer (PreOmics) for 10 min at 95 °C, centrifuged at 1000 rpm, and then treated as described in a previous work.^41^ The resulting peptides were analyzed by a nano-ultra-high performance liquid chromatography tandem mass spectrometry (UHPLC-MS/MS) system using an Ultimate 3000 RSLC coupled to an Orbitrap Fusion Tribrid mass spectrometer (Thermo Scientific Instrument). Elution was performed with an EASY spray column (75 μm × 25 cm, 2 μm particle size, Thermo Scientific) at a flow rate of 400 nL/min using a nonlinear gradient of 2–30% solution B (80% acetonitrile and 20% H_2_O, 5% dimethyl sulfoxide, 0.1% formic acid) in 60 min. MS analysis was performed in DIA mode. Orbitrap detection was used for MS1 measurements at a resolving power of 120 K in a range between 375 and 1500 *m*/*z* and with a 300% AGC target. Advanced Peak Determination (APD) was enabled for MS1 measurements. High-field asymmetric waveform ion mobility spectrometry–compensation voltage (FAIMS CV) was set to −50 at standard resolution and with a total carrier gas flow of 1.5 L/min. Precursors were selected for data-independent fragmentation with an isolation window width of 25 *m*/*z* in 24 windows ranging from 380 to 980 *m*/*z*, with a 2 *m*/*z* overlap. Higher collisional dissociation (HCD) energy was set to 30%, and MS2 scans were acquired at a resolution of 15 k and 100% automatic gain control (AGC) target. All DIA raw files were processed with Spectronaut (version 16)^50^ using a library-free approach (directDIA) under default settings.

The library was generated against the Uniprot Human database (release UP000005640_9606 February 2022). Carbamidomethylation was selected as a fixed modification, and methionine oxidation and N-terminal acetylation were selected as variable modifications. The false discovery rate (FDR) of peptide spectrum match (PSM) and peptide/protein groups was set to 0.01. For quantification, Precursor Filtering was set to Identified (Qvalue), and MS2 was chosen as quantity MS-level. The Protein Quant Pivot Report generated by Spectronaut was statistically evaluated using Perseus software (version 1.6.15.0).^51^ GO enrichment specific for the prostate gland was obtained with the Web server ShinyGO (version 0.75).

### 2.6 Statistical Analysis

Data are expressed as mean ± standard deviation (SD). The numbers in parentheses of the numerical results obtained by the Pawley method in the XRD pattern simulation indicate the estimated standard deviation of the last significant digit. All cellular experiments were independently performed in triplicate. The results were analyzed with a one-way analysis of variance (ANOVA) through *R* software (version 4.2.0, R Foundation) followed by Tukey’s honest significant difference (HSD) *posthoc* test. “*” indicates the level of significance, corresponding to *p* < 0.05.

## 3 Results and Discussion

### 3.1 Microstructural, Morphological, and Magnetic Characterization

**Figure 2:**
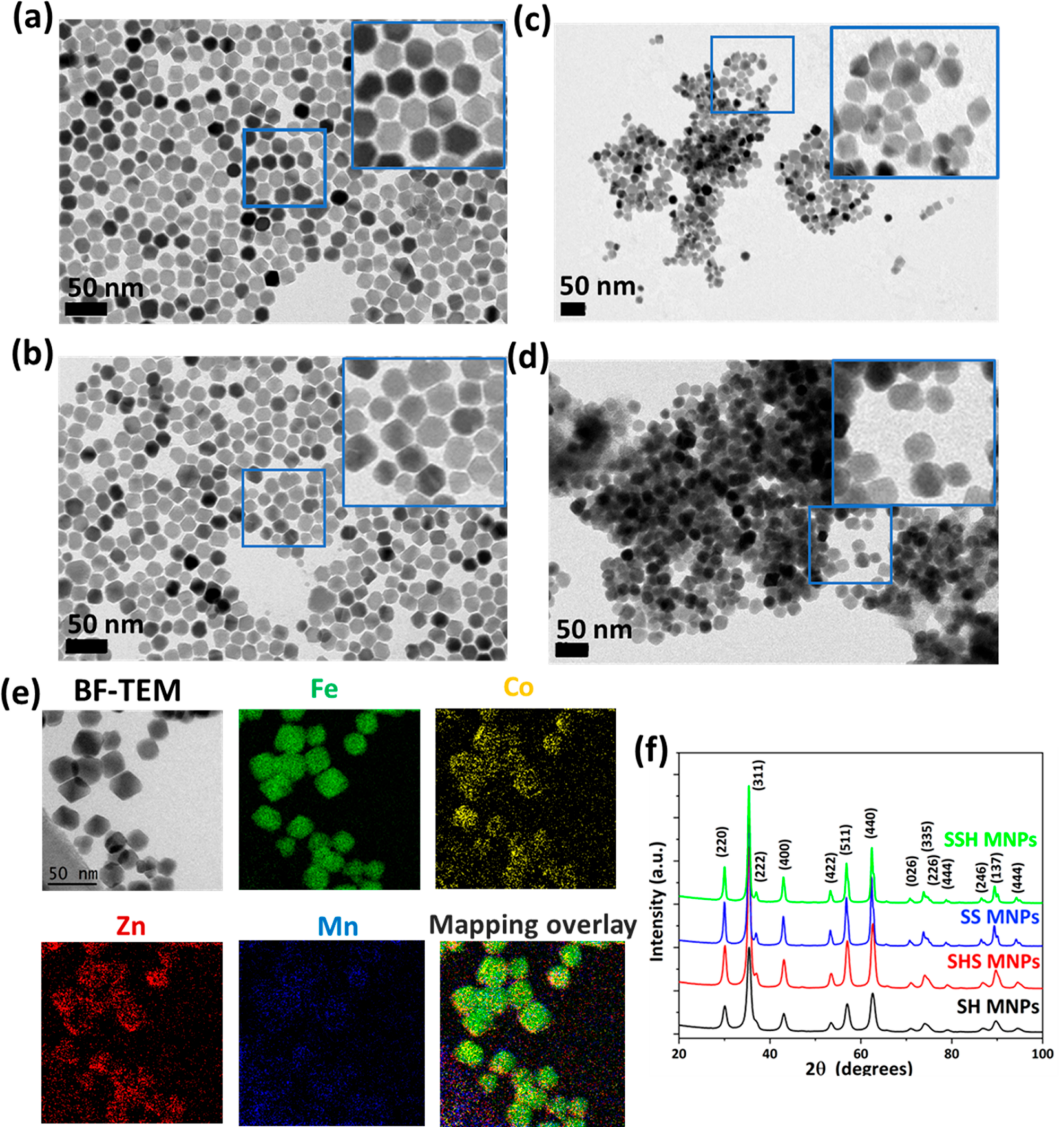
Representative BF-TEM images of (a) SH, (b) SHS, (c) SS, and (d) SSH MNPs. The insets represent the magnified TEM image of corresponding samples. (e) BF-TEM image and corresponding EFTEM elemental maps for the SSH sample. EFTEM mapping demonstrates the presence of Fe, Zn, Mn, and Co. (f) X-ray diffraction patterns of pristine samples (SH, SHS, SS, and SSH MNPs).

Representative transmission electron microscopy (TEM) micrographs of SH, SHS, SS, and SSH MNPs are depicted in Figure 2a–d. The images reveal the cube-octahedral shape of SH nanoparticles (Figure 2a) with a mean diameter of 19.2 ± 1.2 nm (Figure S2a, Supporting Information). SHS TMNPs show similar shapes (Figure 2b) to the seeds, but with an increase in the average size (21.9 ± 0.8 nm) as a result of the coating process (Figure S2b, Supporting Information). Therefore, in the case of SS and SSH MNPs (Figure 2c–d), TEM images show a cube-octahedral morphology of particles. The size distribution histograms (Figure S2c–d, Supporting Information) show mean diameter values of 20.6 ± 0.5 nm (SS) and 23.0 ± 0.8 nm (SSH), confirming the growth of the second shell with approximately 1.5 nm thickness.

To demonstrate the formation of the shell, we mapped the energy distribution of inelastically scattered electrons within the samples using EFTEM. The EFTEM elemental mapping of the SSH (Figure 2e) sample reveals the core–shell–shell nature of the nanoparticles. The results show a Fe-rich signal in the entire nanoparticle that predominantly belongs to the Fe_3_O_4_ core and the distribution of Mn, Zn, and Co atoms located in the Mn_0.5_Zn_0.5_Fe_2_O_4_ and CoFe_2_O_4_ shells, respectively. Similar data have been obtained for the magnetic core–shell architecture of SS MNPs (Figure S3, Supporting Information).

XRD patterns show that SH, SHS, SS, and SSH samples are highly crystalline, and no presence of a residual phase has been observed (Figure 2f). The main diffraction peaks were indexed to planes (220), (311), (222), (400), (422), (551), (440), (335), and (137) and matched to a face-centered cubic (fcc) unit cell of the spinel ferrite with the *Fd*3*m* space group (ICSD file no. 170911, PDF-4 Database).

The mean crystallite size of nanoparticles for each sample is slightly smaller than the average diameter obtained from TEM measurements (Table S1, Supporting Information) and may be explained by the existence of amorphous surface layers.^52^ The lattice parameter (*a*) of the cubic unit cell for each sample (Table S1, Supporting Information) is very close to the magnetite (*a* = 8.380 Å).^31^ We note a slight decrease in lattice constant for SHS and SSH samples compared to SH and SS samples, respectively, which can be attributed to the lattice mismatch between the seed and the outermost layer but that also may be due to lattice distortion or internal stress.^53^

The cube-octahedral morphology of SH and SHS MNPs, respectively, and their nanoscale arrangement is clearly perceived in SEM images (Figure S4a–b, Supporting Information). The images reveal a regular arrangement of SHS MNPs with an average edge length of 23 nm with smooth faces and cut edges (Figure S4b, Supporting Information). Comprehensive images of the morphology of SS and SSH MNPs are reported in Figure S4c–d, respectively (Supporting Information): a typical micrograph of the SSH nanoparticles shows well-defined faces, truncated edges, and an average edge length of 25 nm (Figure S4d, Supporting Information).

XPS was carried out to investigate the valence of the cations, the surface chemical composition of the pristine MNPs (SH, SHS, SS, SSH), and the magnetic shell formation onto the nanoparticles. The wide scan spectra (Figure S5, Supporting Information) display the typical photoemission peaks from the core levels of the elements composing the pristine MNPs (Fe, Mn, Co, Zn, O, and C), as well as their Auger peaks. High-resolution XPS scans on Fe 2p, Mn 2p, Co 2p, and Zn 2p peaks (Figure S6, Supporting Information) were collected and analyzed to extract detailed information on the chemical states of the metal cations.

Fitting of the transition metals’ XPS 2p spectra often requires particular care, as final-state effects might induce, in addition to usual spin–orbit coupling, shakeup and plasmon loss features, the so-called multiplet splittings, that need to be considered for a proper deconvolution of the peaks.^54−57^ Specifically, multiplet splitting, which is present only for high spin species, requires that each of the two spin–orbital components of a 2p doublet is fitted with a certain number of subcomponents, whose structure in terms of shape, full-width-at-high-maximum (fwhm), binding energy (BE) position, and relative intensity depends on the chemical state of the element. The presence of multiple peaks can often be misinterpreted as differing oxidation states.

For these subcomponents, the parameters reported by Biesinger et al.^58^ were used to fit Co 2p, Mn 2p, and Fe 2p regions, while Zn 2p does not present multiplet splitting effects but only the usual spin–orbital splitting into two components.^59^ The fitting of the data, detailed in the Supporting Information, allowed us to identify the oxidation states of the cations in the pristine MNPs. In particular, the best fitting suggests that Co, Mn, and Zn are all present in +2 state, whereas Fe is mainly in +3 state, with a minor contribution from Fe(II). Moreover, we also estimated via XPS the elemental composition of the four pristine samples. The results are reported in Table 1. The experimental formula of SH, SS, SHS, and SSH MNPs was then calculated and compared to the expected composition of the external shells, as XPS probing depth is typically in the 5–10 nm range.^60^

**Table 1:** Elemental composition of Pristine Samples (SH, SHS, SS, and SSH MNPs) Determined by XPS

MNP sample	Co (%)	Fe (%)	Mn (%)	Zn (%)	O (%)	Theor. formula (external shell)	Exp. formula (external shell)
SH	8.5	30.9	0	5.5	55.1	Co_0.5_Zn_0.5_Fe_2_O_4_	Co_0.6_Zn_0.4_Fe_2.2_O_4_
SHS	0.9	32.1	5.7	0.4	60.8	MnFe_2_O_4_	Co_0.06_Zn_0.03_Mn_0.4_Fe_2.1_O_4_
SS	0	22.8	10.3	12.9	53.9	Mn_0.5_Zn_0.5_Fe_2_O_4_	Mn_0.8_Zn_1_Fe_1.7_O_4_
SSH	7.9	30.7	0.9	0.9	59.5	CoFe_2_O_4_	Mn_0.06_Zn_0.06_Co_0.5_Fe_2.1_O_4_

The obtained composition for the SH sample is very similar to the theoretical formula for the shell, suggesting its formation. The slight excess of Fe can be attributed to a fraction of the signal coming from the Fe_3_O_4_ core of the system. For the SHS system, a lower Mn/Fe atomic ratio than the expected value for the external shell has been observed. This result may be explained by the partial leaching of the Mn cations out of the shell layer.^31^ Furthermore, the presence of Zn and Co signals, associated with the inner shell, suggests that the outer layer is very thin (thickness ≈ 1.7 nm). In the case of the SS sample, we note a lower amount of Fe and a higher amount of Zn and Mn with respect to the theoretical prediction, which may be attributed to the cationic diffusion mechanism or to the formation of an undesired Zn- and Mn-rich phase. Related to SSH nanoparticles, a lower amount of Co was detected in the external shell, but the presence of Mn and Zn atoms from the inner shell validates the formation of a very thin outer layer with the thickness under the sampling depth of the XPS beam (∼5 nm).

The XPS compositional analysis of pristine samples has been compared to the ICP-OES results (Table S2, Supporting Information), which should provide a closer estimation of the composition of the whole volume of the MNPs. The Fe content evaluated by ICP-OES is higher than that obtained by XPS for all the samples, as a result of the core–shell structure of the nanoparticles. Accordingly, the higher Co and Zn amounts (for sample SH) and Mn and Zn amounts (for sample SS) observed via XPS with respect to ICP support the shell formation around the magnetite core in both SH and SS samples. The XPS-ICP comparison is less straightforward in the core–shell–shell nanoparticles; however, the ICP and XPS results suggest that Mn is prevalently on the surface of the SHS system. Similar considerations are valid for SSH samplse, with Co prevalently located on the surface.

**Figure 3:**
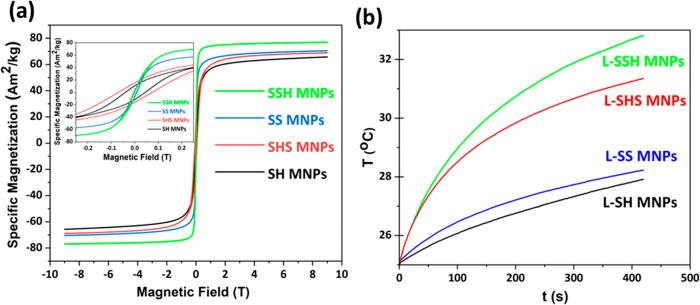
(a) Magnetic curves at room temperature of pristine samples (SH, SHS, SS, and SSH MNPs). The inset evidences the coercivity values. (b) Heating profile of ferrofluid samples (L-SH, L-SHS, L-SS, and L-SSH MNPs) exposed to AMF (*f* = 97.5 kHz, *B* = 20 mT, *H* = 15.9 kA/m; MNP concentration 5 mg/mL).

The magnetization curves of the pristine samples are presented in Figure 3a. The shape-anisotropic nanoparticles envisage a superparamagnetic behavior for SS and SSH samples and nonzero coercivity in the case of SH and SHS, respectively (inset of Figure 3a).

In both cases (SSH and SHS), we noted a slight increase in specific saturation magnetization (*M*_s_) for TMNPs compared to their bimagnetic counterparts (SS and SH samples, respectively; Table S3, Supporting Information). The specific magnetization of SSH MNPs (*M*_s_ = 77.1 Am^2^/kg) is higher than that of the SHS MNPs (*M*_s_ = 68.2 Am^2^/kg; Figure 3a). These results are different than those regarding bimagnetic nanoparticle systems (CoFe_2_O_4_@ MnFe_2_O_4_ and MnFe_2_O_4_@ CoFe_2_O_4_), where *M*_s_ is relatively similar.^61^ We note that the physical phenomena ruling the magnatic properties of TMNPs are more complex than BMNPS and should be explored more extensively considering parameters such as shell thickness, nature of materials, or intermediate layer interdiffusion.^62^ The magnetic remanence (*M*_r_ = 11.7 Am^2^/kg) of SHS MNPs is higher with respect to that of the SSH MNPs (*M*_r_ = 0.55 Am^2^/kg); moreover, the coercivity is notably different in the two systems of TMNPs. The magnetic curves show a 10-fold increase in the coercive field of SHS MNPs (*H*_c_ = 33.4 kA/m) with respect to that of SSH MNPs (*H*_c_ = 5.5 kA/m) nanoparticles. Kubisztal et al. have shown the anisotropy constant of CoFe_2_O_4_ nanoparticles (≈ 8 × 10^5^ J m^–3^) is slightly different compared to Co_0.5_Zn_0.5_Fe_2_O_4_ (≈ 6 × 10^5^ J m^–3^) nanoparticles.^63^ The effective magnetic anisotropy and coercive field decrease with the increase of Zn concenteration in Zn_*x*_Co_1–*x*_Fe_2_O_4_ due to the substitution of Co^2+^ ions of the octahedral site by the nonmagnetic Zn^2+^. Thus, taking into consideration a relatively similar thickness of inner and outer layers in each sample of our TMNPs, the difference in their coercivities might be related to the quality of the interfaces between the different materials.^56^

However, we further note a decrease in the coercivity of nanoparticles with SHS architecture with respect to their core–shell counterpart. Hence, the coercive field of SHS nanoparticles is 35% lower with respect to that of the SH system (*H*_c_ = 50.9 kA/m; Table S1, Supporting Information). A similar result was observed when MnFe_2_O_4_@CoFe_2_O_4_ nanoparticles have been coated with an additional shell comprising a soft magnetic phase, i.e., NiFe_2_O_4_.^19^

We evaluated the heating performance of L-SH, L-SHS, L-SS, and L-SSH MNPs using physical parameters within the range of clinical trials, i.e., AMF frequency *f* ≈ 100 kHz and field amplitude *H* = 2.5–18.0 kA/m.^64^ From the calorimetric measurements of all the samples (Figure 3b), we obtained the highest heating ability (SAR = 69.6 W/g) in the case of SSH MNPs which is higher than the value of its corresponding bimagnetic core–shell seed (SAR = 45.1 W/g; Table S3, Supporting Information). Limiting the contributions of the external parameters, the ILP value demonstrated a higher conversion of magnetothermal energy for SSH MNPs (2.82 nH m^2^/kg; Table S3, Supporting Information) in comparison with the commercial magnetite Feridex (0.15 nH m^2^/kg).^65^ The obtained value is about 19-fold higher than the FDA (Food and Drug Administration)-approved Feridex for biomedical applications, revealing the high potential application of SSH MNPs for MFH. A higher SAR was observed for the SHS system compared to the SH system (Table S3, Supporting Information): these data may be mainly described by the surface spin anisotropy of nanoparticles associated with the additional magnetic shell coating of BMNPs.^17^ This layer could enhance the magnetic exchange coupling interactions at the trimagnetic shell–shell interface, resulting in SAR improvement, analogously to the bimagnetic core–shell nanoparticle behavior.^66^

Due to its highest heating efficiency among all studied samples, the SSH system was selected for further studies.

### 3.2 Cytocompatibility Evaluation

Figure S7 (Supporting Information) presents the WST-1 cell viability assay for L-SSH MNPs. The results demonstrated that L-SSH MNPs, up to 250 μg/mL, do not significantly affect the viability of both PC3 cells (Figure S7a) and human primary normal prostate cells (Figure S7b). At higher concentrations (500 μg/mL and 700 μg/mL), a significant decrease in cell metabolic activity was observed in both cell types. All the subsequent tests have been thus performed using the highest safe concentration of L-SSH nanoparticles (250 μg/mL).

Compared to other superparamagnetic nanostructures, L-SSH MNPs showed a biocompatibility level that is comparable to that of superparamagnetic iron oxide nanoparticles (SPIONs). Indeed, SPIONs showed a safe concentration of 100 μg/mL in SCC-9 cells derived from a primary tumor of the tongue when coated with human serum albumin^67^ and of 400 μg/mL in the U87 glioblastoma-derived cell line when encapsulated in lipids.^18^ Since SPIONs are considered safe nanomaterials and have been approved in clinics, the cytocompatibility observed for L-SSH is satisfying. However, the stability and safety of the nanoparticles should be confirmed in future experiments *in vivo*, where the degradation of the external layers of the nanostructure may expose cells to Co and thus induce toxicity.

### 3.3 Characterization of L-SSH, CM-L-SSH, LN1-L-SSH, and CM-LN1-L-SSH MNPs

This study aims at developing a novel strategy with an optimized heating efficiency for magnetic-mediated intracellular hyperthermia, along with improved stability, biocompatibility, and selective targeting of PCa cells, that potentially minimize systemic toxicity and maximize therapeutic benefit. Thus, the L-SSH system was further conjugated with LN1 cell-penetrating peptide (LN1-L-SSH MNPs), coated with cell membranes (CM-L-SSH MNPs) or functionalized with both CM and LN1 (CM-LN1-L-SSH MNPs). LN1 was preferred instead of antibodies, which commonly are much larger molecules, due to its high binding avidity and selectivity for PCa cells.^30^ It is also known that it may induce apoptosis with high selectivity toward prostate cancer cells.^30^ Cell-membrane-coated nanoparticles, on the other hand, can avoid protein adsorption and phagocytosis by the reticuloendothelial system (RES), extending their circulation time *in vivo*.^68^

**Figure 4:**
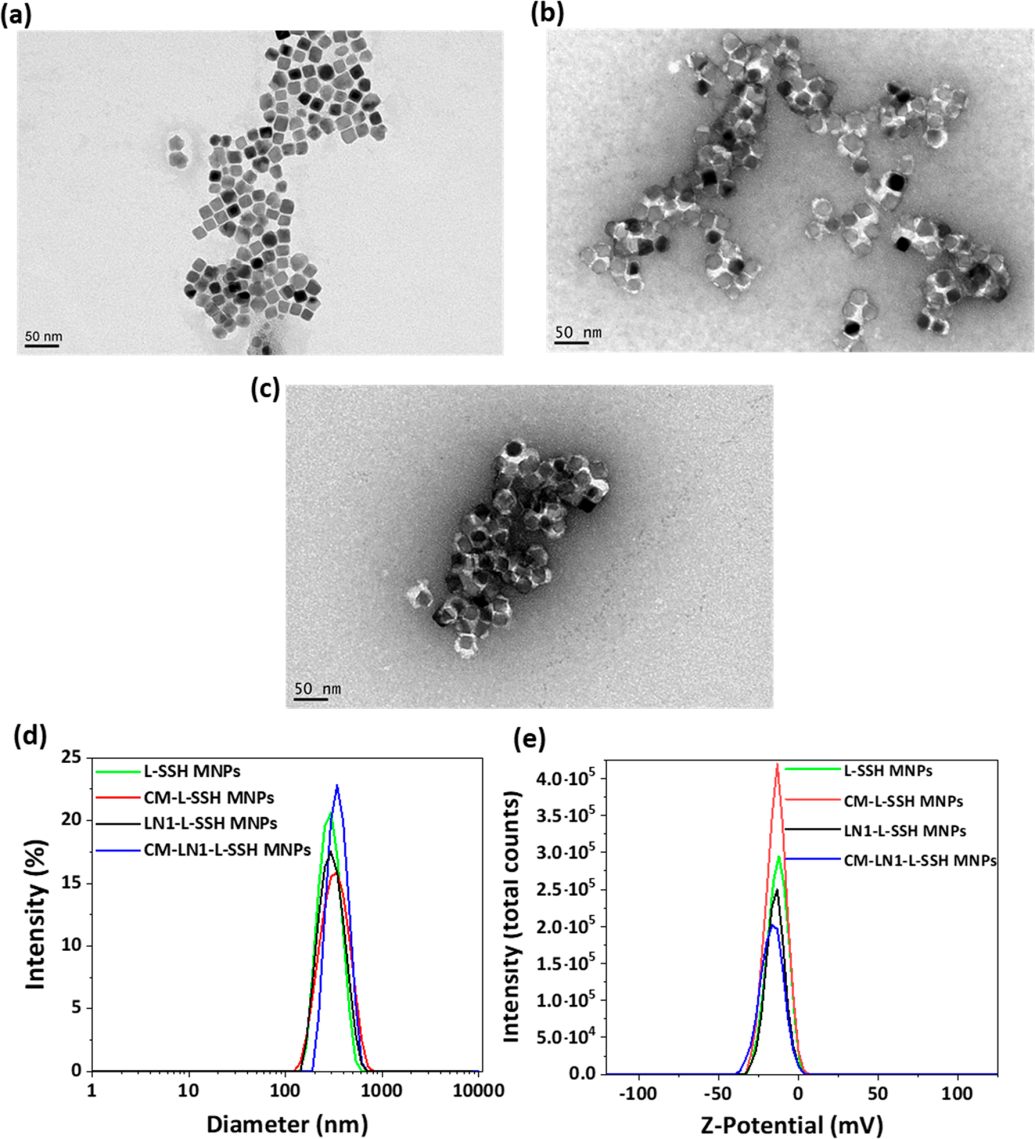
BF-TEM micrographs of functionalized samples: (a) LN1-L-SSH MNPs, (b) negative-stained CM-L-SSH MNPs, and (c) negative-stained CM-LN1-L-SSH MNPs. DLS measurements: (d) hydrodynamic size distribution and (e) *Z*-potential before (L-SSH MNPs) and after functionalization (CM-L-SSH, CM-LN1-L-SSH, and CM-LN1-L-SSH MNPs).

The TEM micrographs reported in Figure 4a–c show the representative morphology of functionalized samples: LN1-L-SSH, CM-L-SSH, and CM-LN1-L-SSH MNPs, respectively. Figure 4a reveals the LN1-conjugated TMNPs, while Figure 4b–c confirm the lipid bilayer shell formation of approximately 3–5 nm thickness around the magnetic cores. This result is in agreement with other studies that report an outer thickness of about 5 nm of CM-coated Fe_3_O_4_.^69^ In both cases, the micrographs of CM-L-SSH and CM-LN1-L-SSH MNPs present aggregates of magnetic nanoparticles with an inorganic core well encapsulated within the lipid coating.

The DLS analysis of MNPs (Figure 4d) has shown an increasing trend in the mean hydrodynamic diameter from 293 ± 8 nm (L-SSH MNPs) to 334 ± 12 nm (CM-LN1-L-SSH MNPs) (Table S4; Supporting Information). The mean hydrodynamic diameter of LN1-L-SSH MNPs and CM-L-SSH MNPs is 304 ± 6 nm and 329 ± 6 nm, respectively. The polydispersity index (PDI) for each sample is reported in Table S4 (Supporting Information). After CM coating, a slight decrease of *Z*-potential (Figure 4e) from −12.4 ± 0.6 mV (L-SSH MNPs) to −13.9 ± 0.2 mV (CM-L-SSH MNPs) and to −14.7 ± 0.2 mV (CM-LN1-L-SSH MNPs) suggests an enhancement of colloidal stability (Table S4, Supporting Information). Other researchers claimed a similar range of *Z*-potential (−18 mV to – 13 mV) when myeloid-derived CM is used to coat Fe_3_O_4_ nanoparticles.^27^ A slightly lower value of the *Z*-potential (−11.8 ± 0.7 mV) for LN1-L-SSH MNPs was finally determined (Table S4, Supporting Information).

The CM-LN1-L-SSH MNP stability has been evaluated mimicking various physiological conditions using PBS, DMEM, and 90% DMEM + 10% FBS (Figure S8, Supporting Information): the nanoparticles remain stable over 1 month and demonstrated a slightly lower stability than L-SSH MNPs.

TGA measurements were performed to quantify the amount of organic fraction grafted onto SSH MNPs as well as the magnetic phase present in the specimens (Figure S9, Supporting Information). The initial weight loss of about 1.4% occurring in the temperature range 80–100 °C may be assigned to the loss of water molecules adsorbed on the surface of the nanoparticles. The weight plot along increasing temperature of functionalized samples (CM-SSH, LN1-L-SSH, CM-LN1-L-SSH MNPs) suggests that the thermal degradation of the lipid component occurs in the range 190–280 °C. This temperature range matches very well with the endothermal decomposition of the short chain of amino acids.^70^ The main peak observed on the derivative thermogram (DTG) of each sample ranges from 315 to 370 °C, and it is attributed to thermal degradation of the PEG component. The peak shift to lower temperatures in the first derivative plot may be due to competitive processes and various intramolecular reactions during the combustion of the cross-linked network (e.g., chain mobility during gelation), to the coverage density of PEG chains at the nanoparticle surface, or to the heat resistance of the PEG crystalline phase.^71,72^ The organic mass loss of each sample during TGA (L-SSH MNPs: 6.7%, LN1-L-SSH MNPs: 7.4%, CM-L-SSH MNPs: 7.7%, CM-LN1-L-SSH MNPs: 9.3%; Table S4, Supporting Information) is in excellent agreement with the specific functionalization procedure, evidencing an efficient coating process of MNPs. As the ferrites are highly stable until 600 °C, the remaining mass represents the percentage of magnetic phase in the samples: 93.3% (L-SSH MNPs), 92.3% (CM-L-SSH MNPs), 92.6% (LN1-L-SSH MNPs), and 90.7% (CM-LN1-L-SSH MNPs) (Table S3, Supporting Information). The increasing trend of weight loss after 450 °C may be assigned to the adsorption of N_2_ molecules at the nanoparticle surface.

The BCA assay revealed that the amount of LN1 peptide in 1 mg/mL of LN1-L-SSH MNPs is 11.2 ± 3.6 μg/mL. The protein amount in 1 mg/mL of CM-L-SSH MNPs is instead 134.7 ± 10.2 μg/mL. Interestingly, 1 mg/mL of CM-LN1-L-SSH MNPs is characterized by a protein amount of 173.3 ± 1.2 μg/mL, a value higher than the simple sum of the values for LN1-L-SSH MNPs and CM-L-SSH MNPs: this increment can be attributed to the improved LN1 functionalization efficiency when used in combination with CM.

We therefore evaluated the magnetic behavior and heating efficiency of CM-LN1-L-SSH MNPs (Figure S10a, Supporting Information). The results show that CM-LN1-L-SSH MNPs did not change the superparamagnetic behavior (*H*_c_ = 6.3 kA/m), whereas the saturation magnetization (*M*_S_ = 66.2 kA/m^2^) is lower than that of the pristine sample (*M*_S_ = 77.1 kA/m^2^), mainly because of the organic coating. Therefore, the magnetothermal experiment demonstrated a decrease of SAR from 69.6 W/g (L-SSH) to 61.2 W/g (CM-LN1-L-SSH MNPs; Figure S10b, Supporting Information).

### 3.4 Cellular Internalization

**Figure 5:**
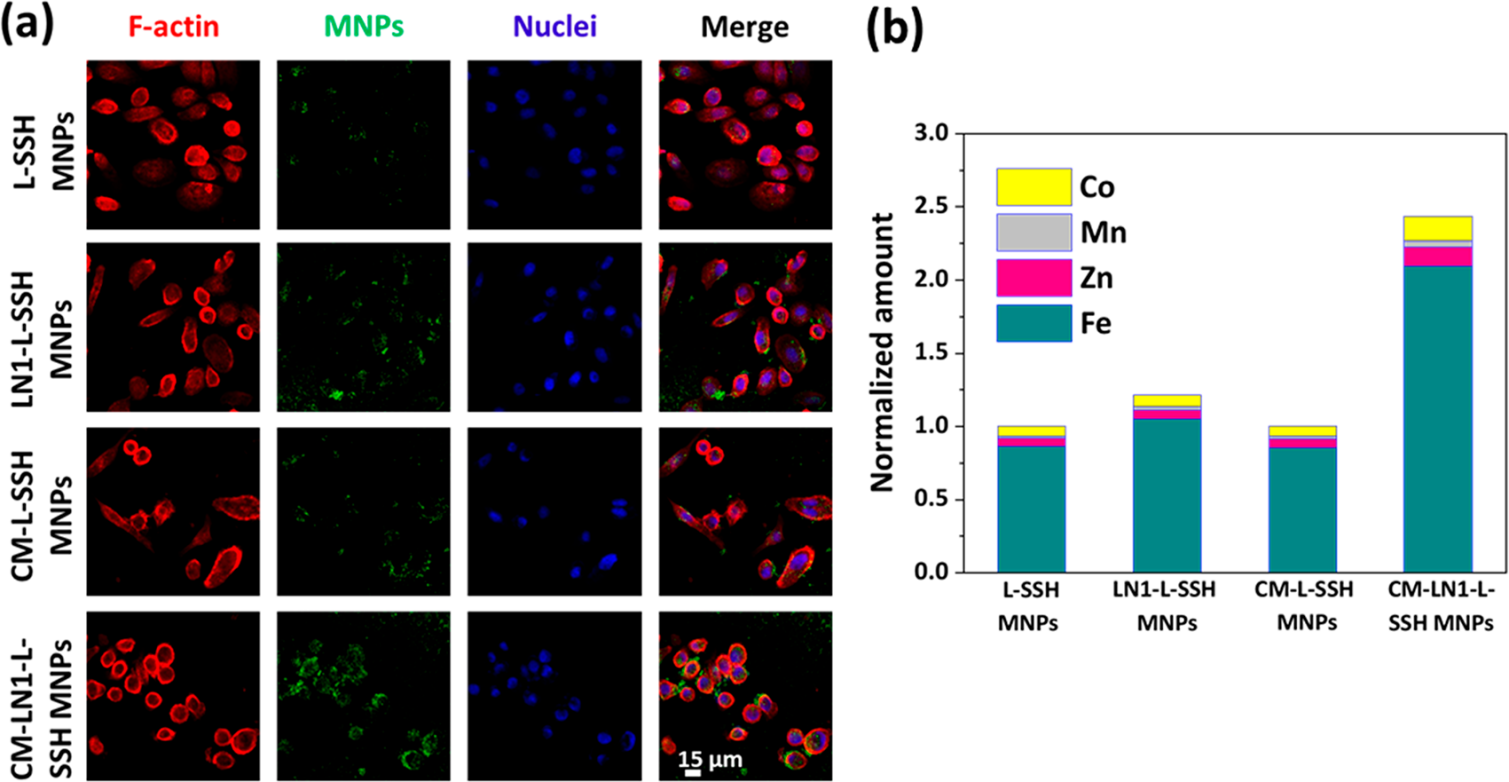
(a) Representative confocal images of PC-3 cultures incubated for 72 h with 250 μg/mL of MNPs (L-SSH, LN1-L-SSH, CM-L-SSH, and CM-LN1-L-SSH MNPs). F-actin in red, MNPs in green, and nuclei in blue. (b) ICP-OES elemental quantification of Fe (green), Zn (pink), Mn (gray), and Co (yellow) in PC-3 cells treated with the different MNPs.

Figure 5a depicts representative images of performed analyses, and it shows F-actin (in red), MNPs (in green), nuclei (in blue), and merged signals. An increase in nanoparticle uptake in the case of LN1-L-SSH and CM-LN1-L-SSH MNPs, with respect to the other experimental classes (L-SSH and CM-L-SSH MNPs), can be appreciated. The localization of MNPs is mainly perinuclear.

The quantitative ICP-OES analysis confirmed the qualitative results obtained by confocal imaging (Figure 5b). A significant increase in cell uptake was found when using LN1-L-SSH nanoparticles compared to L-SSH MNPs (1.2-fold), while the highest uptake efficiency of nanoparticles in PC-3 cells (particularly, in terms of Fe) was observed when using CM-LN1-L-SSH MNPs (2.4-fold compared to L-SSH MNPs).

This result demonstrates that combining the homotypic CM coating approach with LN1 functionalization allows for enhanced targeting. The improved uptake may be attributed not only to the combination of the two targeting approaches but also to the higher amount of LN1 present in the CM-LN1-L-SSH system compared to LN1-L-SSH MNPs. For this reason, CM-LN1-L-SSH MNPs were used for all the subsequent experiments.

### 3.5 Proliferation, Apoptosis, and Necrosis in Response to Magnetothermal Treatment

**Figure 6:**
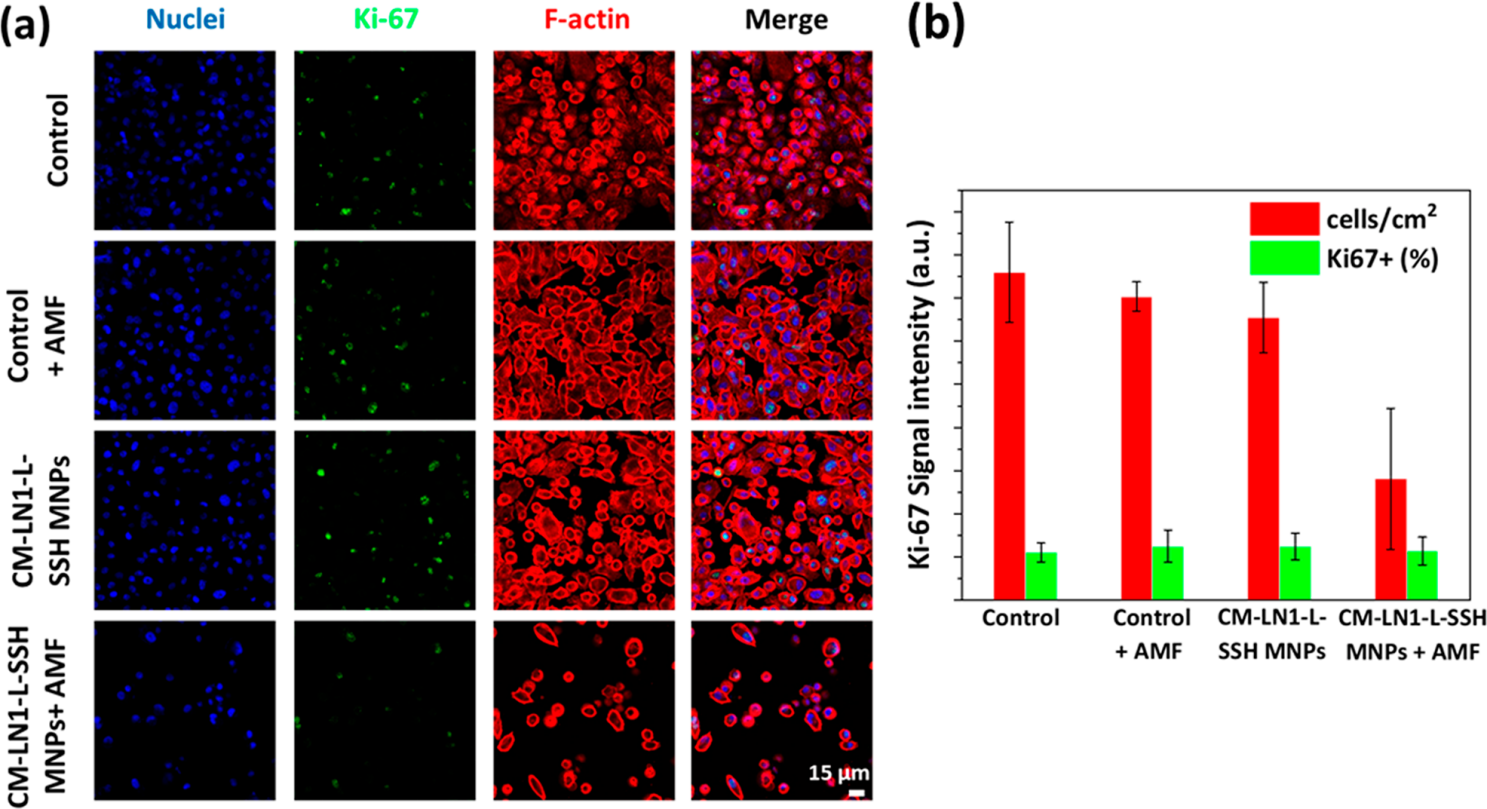
(a) Representative confocal laser scanning microscopy imaging of *K*_i_-67 expression in PC-3 cells in the considered experimental classes. Nuclei in blue, *K*_i_-67 in green, and F-actin in red. (b) In red, % of cells normalized to controls. In green, % of *K*_i_-67-positive cells (*K*_i_-67+).

The effects of hyperthermia treatment following CM-LN1-L-SSH MNP administration have been evaluated. Figure 6a shows the CLSM analysis of the expression of the *K*_i_-67 proliferation marker in the following experimental classes: “Control”, “Control + AMF”, “CM-LN1-L-SSH MNPs”, and “CM-LN1-L-SSH MNPs + AMF”.

To locally investigate the temperature reached by the nanoparticles at the cellular level, the CM-LN1-L-SSH MNPs were stained with the temperature-sensitive lipophilic DiI dye, and CLSM time-lapse imaging was carried out (Figure S11a, Supporting Information). The fluorescence intensity of the CM-LN1-L-SSH MNPs was monitored in real-time during AMF stimulation (Figure S11b). The decrease in fluorescence intensity induced by CM-LN1-L-SSH MNPs + AMF can be attributed to an increase in temperature as described by the Δ*F*/*F*_0_ = −0.0224·Δ*T* equation.^49^ The *T* graph during magnetothermal stimulation is shown in Figure S11c, suggesting that CM-LN1-L-SSH MNPs reach *T* > 40 °C in the cells upon AMF stimulation.

We observed a significant decrease in cell density in response to the “CM-LN1-L-SSH MNPs + AMF” treatment (36.9 ± 21.4%) compared to the other experimental classes (100.0 ± 15.3% for “Control”, 92.5 ± 4.4% for “Control + AMF”, 86.1 ± 10.7% for “CM-LN1-L-SSH MNPs”; Figure 6b). Also, a lower *K*_i_-67 expression signal intensity (in green) was observed in the “CM-LN1-L-SSH MNPs + AMF” class compared to the other experimental groups (Figure 6a). However, despite the lower signal intensity, a similar % of *K*_i_-67^+^ cells (Figure 6b) was found in the “CM-LN1-L-SSH MNPs + AMF” treatment (24.0 ± 4.7%) compared to “Control” (27.2 ± 8.1%), “Control + AMF” (24.8 ± 6.9%), and “CM-LN1-L-SSH MNPs” (27.0 ± 4.7%). Considering that the same cell number was seeded in the different experimental classes, the result indicating a lower number of cells upon magnetothermal stimulation can be attributed to cell death and detachment phenomena, and it is in line with the WST-1 assay (Figure S12, Supporting Information), where a remarkable decrease in cell viability was detected in response to the “CM-LN1-L-SSH MNPs + AMF” treatment (54.4 ± 2.3%) but not in the case of “Control + AMF” (90.4 ± 1.6%) and of “CM-LN1-L-SSH MNPs” (103.3 ± 11.1%) treatment. Altogether, the immunofluorescence and WST-1 analyses indicate that the chronic magnetothermal stimulation induced by the AMF treatment in the presence of CM-LN1-L-SSH MNPs produces a significant cytotoxic effect, remarkably reducing the number of cells without using any chemotherapic drug. Also, the stimulation approach has been carried out at safe nanoparticle concentrations and AMF doses. Indeed, no significant effect on cell density was found in the “Control + AMF” and “CM-LN1-L-SSH MNPs” experimental conditions. This chemotherapy-free approach allows us to localize the anticancer treatment only where nanoparticles are accumulated and AMF is applied, thus potentially limiting the side effects of anticancer therapy on healthy tissues.

Concerning the anticancer mechanisms of the “CM-LN1-L-SSH MNPs + AMF” treatment, the decrease in the *K*_i_-67 signal is evidence of decreased proliferation.^73^ The presence of a lower *K*_i_-67 expression is attributed to the slow yet progressive degradation of this marker during quiescence, while the level of decrease depends on the time that cells remained in the G0 cycle phase. The lower amount of this marker, therefore, indicates that the cells upon “CM-LN1-L-SSH MNPs + AMF” treatment are not in the S, G2, and M proliferation phases, yet exit from the cell cycle.

Compared to other magnetic nanoplatforms in the literature, the *in vitro* anticancer efficacy of CM-LN1-L-SSH MNPs is relatively high. Some authors reported the use of highly pure magnetosome for the treatment of PC3 cancer cells by inductive heating:^74^ the treatment with citric-acid-functionalized magnetosome (M-CA) and AMF (*f* = 195 kHz, *B* = 42 mT) was able to reduce by 35.5% the cell viability with respect to the treatment with only M-CA. In another example, the cell viability of BV2 cells decreased by approximately 30% after magnetic hyperthermia treatment induced by poly(acrylic acid)-coated iron oxide nanoparticles and AMF (*f* = 560 kHz, *H* = 23.9 kA/m).^75^

**Figure 7:**
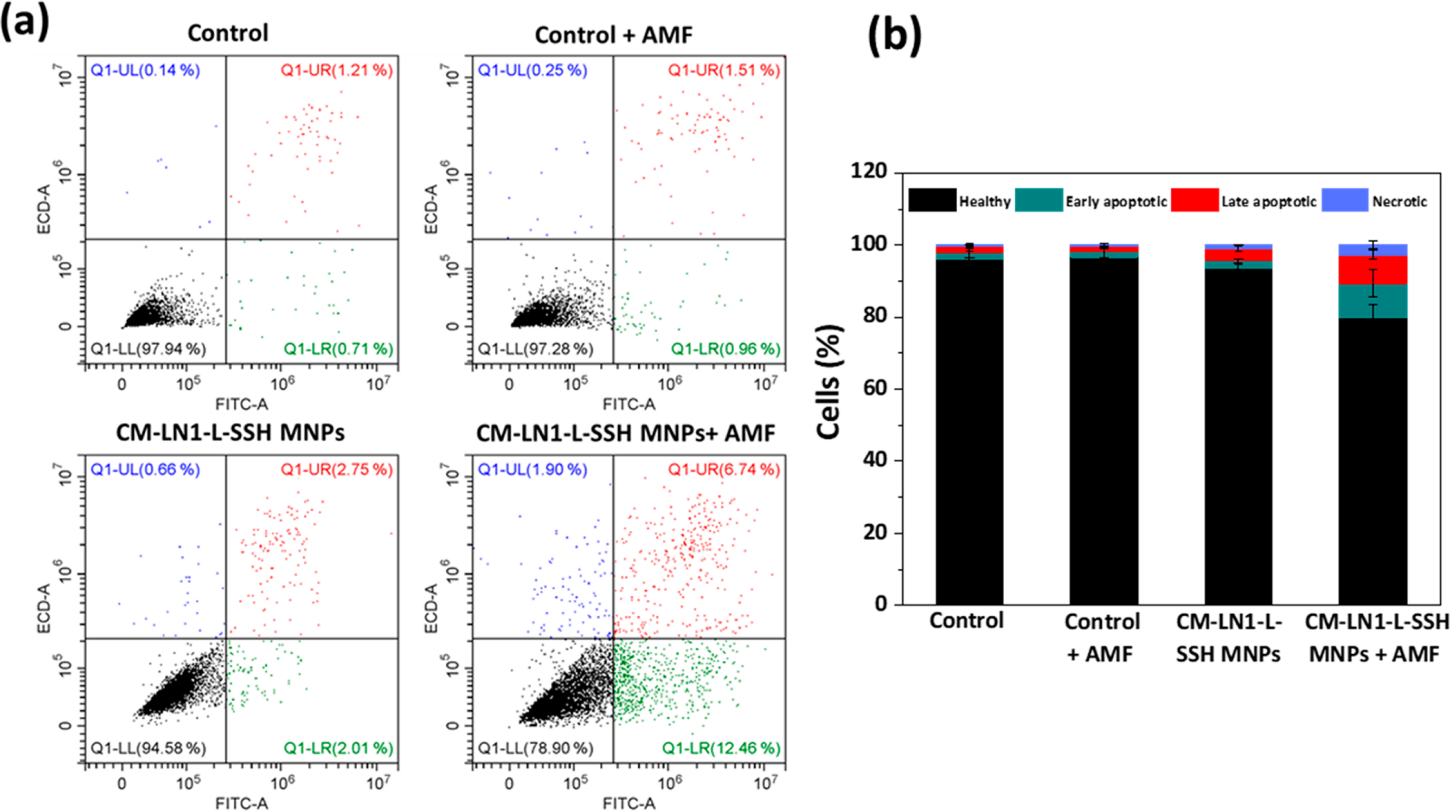
Flow cytometry analysis of apoptosis/necrosis: (a) representative flow cytometer scatter plots of propidium iodide vs annexin V-FITC. The populations of healthy, early apoptotic, late apoptotic, and necrotic cells have been highlighted in black, green, blue, and red, respectively. (b) Quantitative evaluation.

An interesting finding of Calatayud et al. provides that cell viability can still decrease many hours after magnetic treatment due to progressive cell death.^66^ For this reason, apoptosis and necrosis have been investigated. Flow cytometer analysis of early apoptotic, late apoptotic, and necrotic cells (Figure 7) has been carried out to investigate if cell death was involved in the anticancer mechanism induced by the “CM-LN1-L-SSH MNPs + AMF” treatment.

In Figure 7a, representative flow cytometer scatter plots of propidium iodide vs annexin V-FITC are shown, and the populations of healthy, early apoptotic, late apoptotic, and necrotic cells have been highlighted in black, green, blue, and red, respectively. Figure 7b reports the quantitative evaluation of the obtained data. The “CM-LN1-L-SSH MNPs + AMF” treatment induced a significant decrease of the healthy cells (79.8 ± 3.6%) compared to “Control” (96.1 ± 2.1%; *p* < 0.05), “Control + AMF” (96.6 ± 2.6%), and “CM-LN1-L-SSH MNPs” (93.4 ± 1.3%) groups. No remarkable effect on healthy cells was observed in “Control + AMF” and “CM-LN1-L-SSH MNPs” groups compared to “Control”, therefore confirming that the single stimulation does not have harmful effects on PC-3 cells. The decrease of healthy cells in response to magnetothermal stimulation can be attributed mainly to apoptotic phenomena (9.5 ± 3.6% of early apoptotic and 7.9 ± 1.1% of late apoptotic cells in the “CM-LN1-L-SSH MNPs + AMF” group) and, secondarily, to necrotic events (2.7 ± 1.0% in the “CM-LN1-L-SSH MNPs + AMF” group).

We have to highlight that the stimulation time is sufficient to increase the temperature of the nanoparticles in the cells from room temperature to above 40 °C, in the appropriate range of temperatures used for hyperthermia (40–43 °C).^76^ These results indicate that the magnetothermal treatment affects cancer cell viability by both reducing proliferation and inducing cell death primarily through apoptosis. Apoptosis is a programmed cell death that does not induce tissue damage or inflammation. For this reason, traditionally, anticancer treatments inducing apoptosis (e.g., hyperthermia) are preferred with respect to necrosis (e.g., thermal ablation). However, cancer cells can avoid such types of cell death by blocking apoptotic signaling. Overexpression of chaperones, such as heat-shock protein 70 and 27 (hsp70 and hsp27), can inhibit caspase-dependent and caspase-independent apoptotic pathways.^77,78^ These defense mechanisms are normally triggered by cells in response to stress and are particularly developed in cancer cells. Despite the protection offered by chaperones, however, physical treatments (e.g., with electric and thermal cues) are capable of activating apoptotic pathways in cancer cells. In the specific case of thermal stimuli, the temperature of about 42 °C is known to induce apoptosis through the activation of caspases.^79^ Specifically, caspase-9 is highly activated after heat, is downstream of the activation cascade, and represents a good marker of the apoptotic trigger.^69^ For these reasons, experiments have been conducted to analyze the expression of hsp70 and the activation of caspase-9 in PC-3 cells under MNP-mediated hyperthermia.

### 3.6 Biochemical Pathways Activated in Response to Magnetothermal Treatment

**Figure 8:**
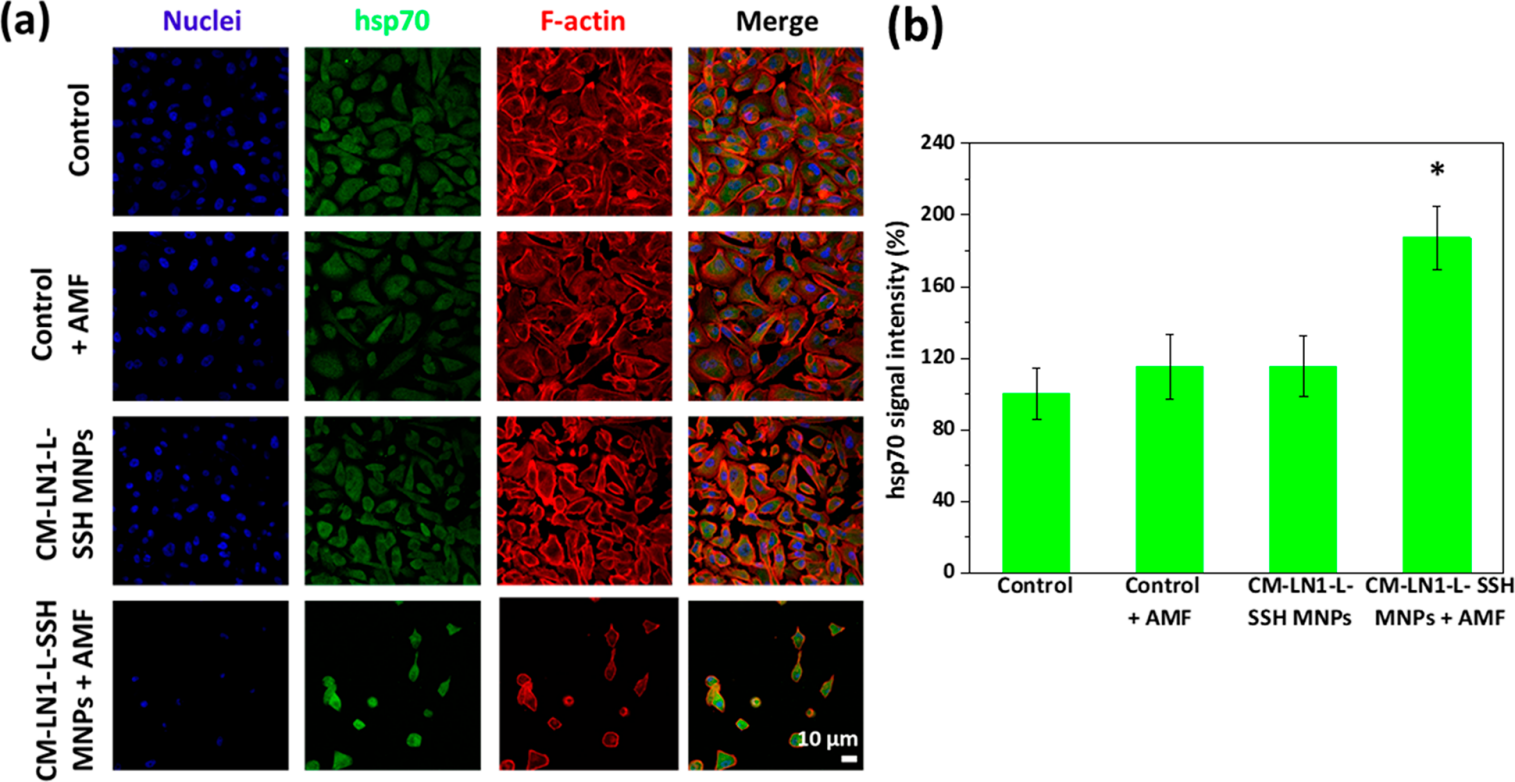
(a) Expression of hsp70 in PC-3 cells upon magnetothermal stimulation: representative confocal laser scanning microscopy imaging (nuclei in blue, hsp70 in green, F-actin in red). (b) Average intensity of the hsp70 signal in the cells for each experimental condition (* *p* < 0.05).

The immunofluorescence analysis of the hsp70 expression in “Control”, “Control + AMF”, “CM-LN1-L-SSH MNPs”, and “CM-LN1-L-SSH MNPs + AMF” experimental classes, 60 min after a single stimulation session with AMF, is shown in Figure 8.

CLSM imaging of the hsp70 expression (in green), F-actin (in red), nuclei (in blue), and the merged signals is presented in Figure 8a. Qualitatively, the low number of cells in the “CM-LN1-L-SSH MNPs + AMF” group that survived to magnetic hyperthermia treatment display a higher hsp70 signal intensity compared to the other experimental classes. In Figure 8b, the graph reports the average intensities of the hsp70 signal in the different experimental classes with respect to “Control”. The hsp70 signal intensity upon magnetothermal treatment (“CM-LN1-L-SSH MNPs + AMF”; 187.2 ± 17.9%) is significantly higher compared to the other experimental classes (100.0 ± 14.2% for “Control”, 115.3 ± 18.1% for “Control + AMF”; 115.3 ± 16.7% for “CM-LN1-L-SSH MNPs”). The increase in hsp70 expression is a well-defined indicator of mild heat stress and is associated with the temperature increase over time.^34^ The observed increase in hsp70 expression can be therefore associated with the cell response to the heat stimulation induced by the AMF in the presence of magnetic nanoparticles. Hsp70 is known to be able to protect cancer cells from apoptosis. The observed enhanced expression of this chaperone in the surviving cells may have reduced the probability to incur heat-dependent apoptotic death. A remarkable hsp70 activation was also observed in ovarian cells treated with magnetic fluid hyperthermia, and different strategies have been tested to inhibit hsp70 expression and function, including siRNA and the 2-phenylethynesulfonamide (PES) inhibitor of hsp70 activity:^80^ these approaches resulted in reduced cell viability in response to the magnetothermal treatment. A synergic combination of magnetic hyperthermia and drugs inhibiting hsp70 can therefore be proposed in future works for enhancing the magnetothermal treatment in prostate cancer as well.

**Figure 9:**
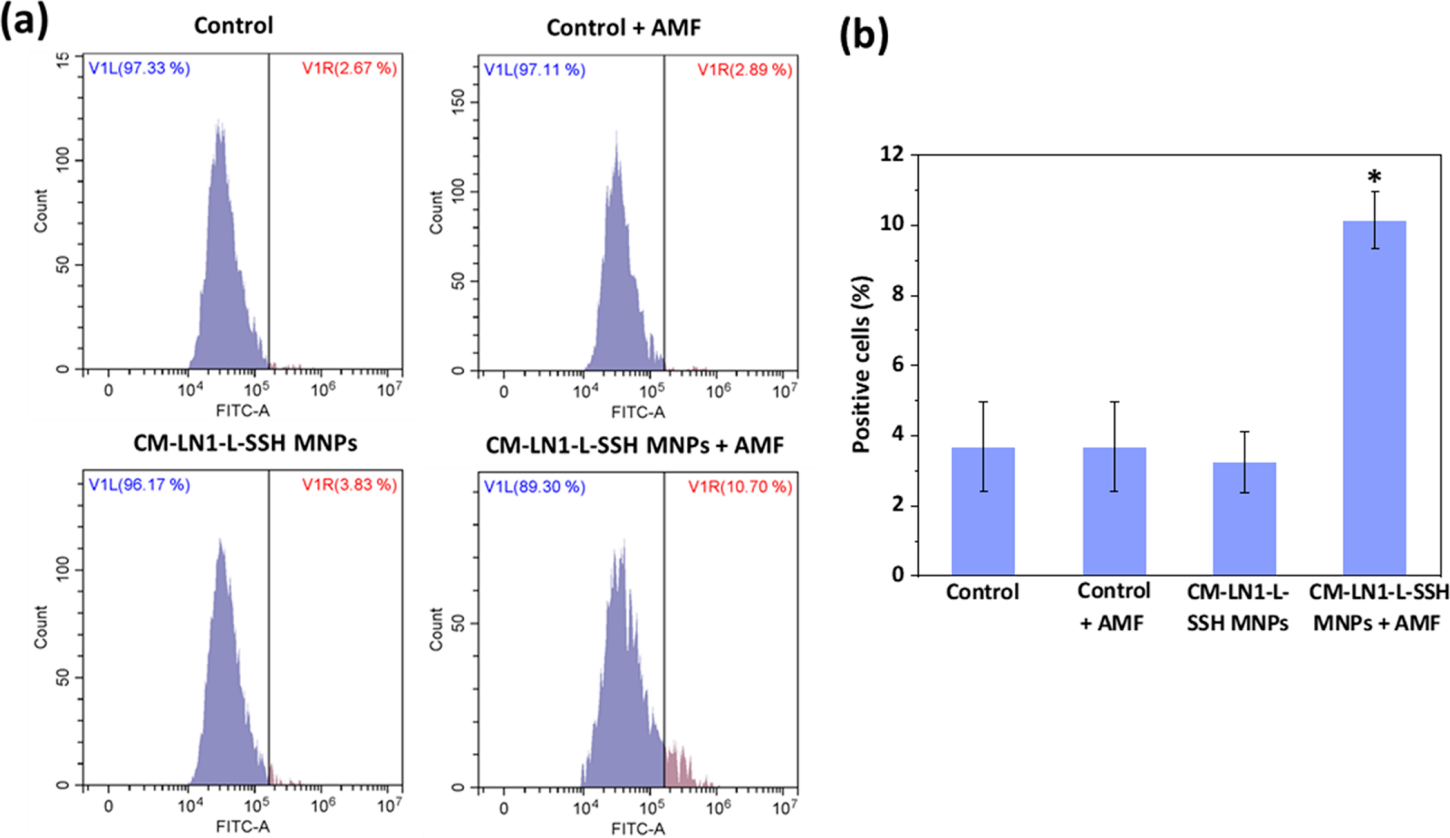
Activation of the caspase-9 apoptotic pathway upon acute stimulation with magnetic hyperthermia (“CM-LN1-L-SSH MNPs + AMF”). (a) Representative distributions of the cell fluorescent signal emission. Caspase-9-negative (−) and -positive (+) cells are highlighted in light blue and light red, respectively. (b) Quantitative evaluation of flow cytometry data for each experimental condition (* *p* < 0.05).

The activation of the caspase-9 apoptotic pathway was investigated in response to the acute stimulation with magnetic hyperthermia (“CM-LN1-L-SSH MNPs + AMF”), and results have been compared to the “Control + AMF”, “CM-LN1-L-SSH MNPs”, and “CM-LN1-L-SSH MNPs + AMF” experimental classes (Figure 9).

Caspase-9 is activated by recruitment and dimerization within the Apaf-1 apoptosome. In turn, once activated, caspase-9 can trigger caspases-3, – 6, and −7 of the apoptotic signal.^37^ The activated form of caspase-9 has been detected by flow cytometry using the FITC-conjugated LEHD-FMK. In Figure 9a, representative graphs with the fluorescence emission distributions of the four experimental groups are presented; in Figure 9b, the graph reports the % of caspase-9^+^ cells. A significant increase of caspase-9^+^ cells has been observed in the “CM-LN1-L-SSH MNPs + AMF” group (10.1 ± 0.8%) with respect to “Control” (3.0 ± 0.4%), “Control + AMF” (3.7 ± 1.3%), and “CM-LN1-L-SSH MNPs” (3.3 ± 0.9%) groups. The reported 3.4-folds increase in the caspase-9 activated form in “CM-LN1-L-SSH MNPs + AMF” compared to “Control” after a single magnetothermal treatment is in agreement with the enhanced apoptotic levels previously reported, suggesting that the triggering of the apoptotic pathway in response to magnetic hyperthermia can be associated with the activation of caspase-9.

### 3.7 Cell Migration upon Acute Magnetothermal Stimulation

**Figure 10:**
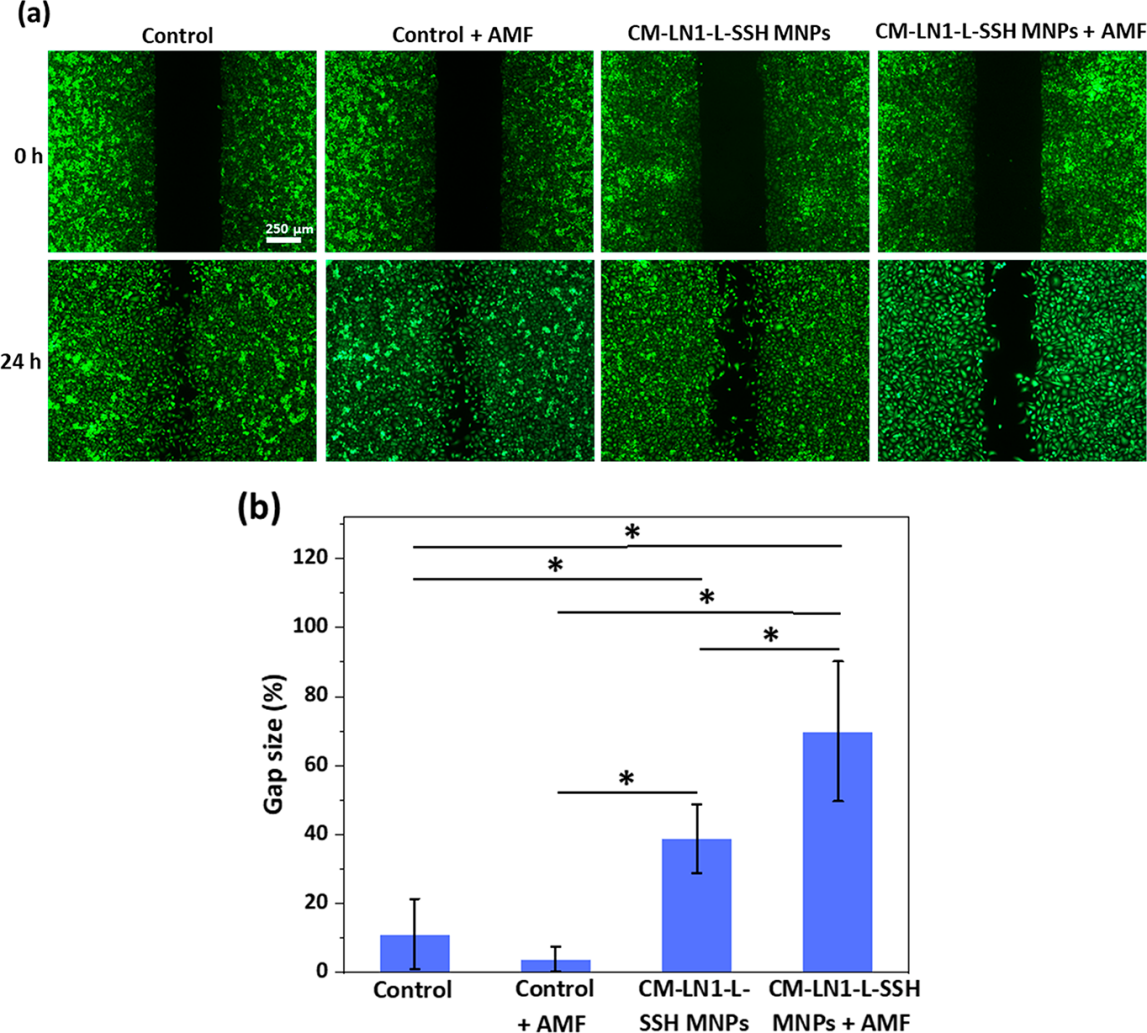
Cell migration upon acute magnetothermal stimulation. (a) Representative images of PC-3 cells stained with calcein at *t* = 0 h and *t* = 24 h of cell migration. (b) % of gap size measured in each experimental class (* *p* < 0.05).

The cell migration ability of PC-3 cells was analyzed *in vitro* in the presence of CM-LN1-L-SSH MNPs and AMF stimulation (Figure 10). The representative fluorescence images of the migration area before and after the stimulation (*t* = 0 h and *t* = 24 h) are shown in Figure 10a, while the percentage of gap size is reported in Figure 10b. The cells treated with CM-LN1-L-SSH MNPs in the presence or absence of AMF stimulation showed a significant reduction in PC-3 cell migration with respect to the control cultures. The gap size increased to 30.9 ± 9.9% and 69.9 ± 20.2% in CM-LN1-L-SSH MNP-treated cells in the presence and absence of AMF stimulation, respectively, while the gap size was 11.2 ± 10.3% and 3.8 ± 3.6% in control cultures. In addition, the CM-LN1-SSH MNP-treated cells showed a significant increase in gap size when AMF was applied, if compared to the only CM-LN1-SSH MNP-treated cultures.

Although metastatic cascade is a complex process in cancer progression, cell migration is a pivotal step for the initiation of long-distance metastasis.^81^ Here, the PC-3 cell migration was evaluated by *in vitro* scratch assay providing information about the migration capacity over time. Some studies suggested that iron oxide nanoparticles restrict the migration of cells by inhibiting the activity of the actin cytoskeleton and destroying microtubule networks, resulting in loss of focal adhesion.^82,83^ In another study, SPIONs decreased the migration of glioblastoma cells by inhibiting the invasion capability of cells via mannose-6-phosphate (M6P) receptors.^84^

Hyperthermia is capable of adversely affecting cell membrane fluidity and stability as well as altering the function of transmembrane proteins and cell surface receptor expression.^85^ Notably, tumor cells are more sensitive to temperature increment than normal cells, which makes hyperthermia an attractive tool for antitumor therapy.^86^ In our study, PC-3 cells reacted to the CM-LN1-L-SSH MNP-mediated hyperthermia by overexpressing the hsp70 protein: recently, a study showed that the overexpression of hsp70 proteins in A549 lung cancer cells leads to an inhibition of TGF-β signaling, which plays a significant role in cell migration reduction,^87^ thus corroborating our findings.

### 3.8 Proteomic and Gene ontology (GO) Analysis

**Figure 11:**
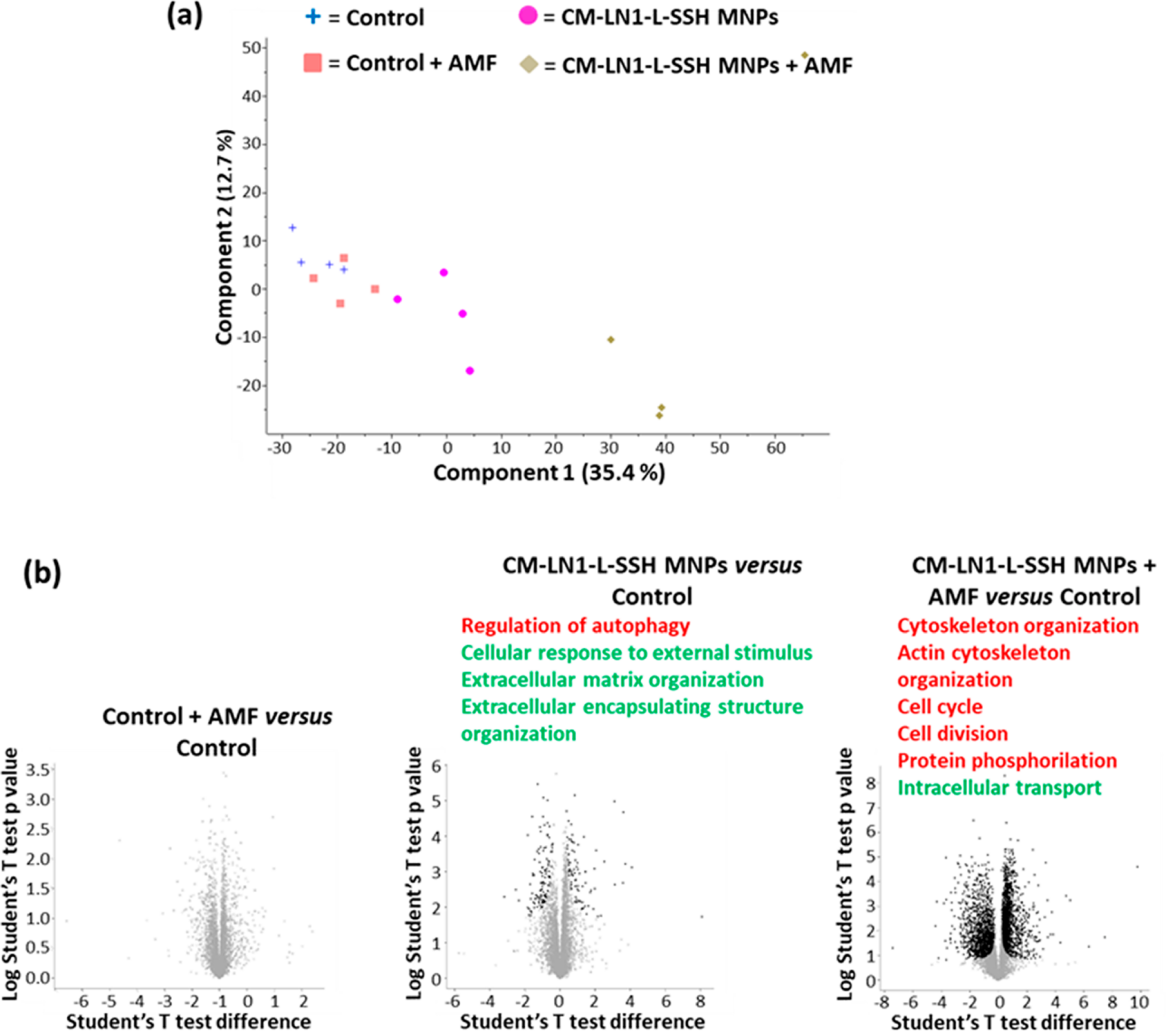
Proteomic analysis: (a) principal component analysis (PCA) for 4 independent experiments in “Control” (blue cross), “Control + AMF” (orange square), “CM-LN1-L-SSH MNPs” (magenta circles), and “CM-LN1-L-SSH MNPs + AMF” (rhombuses in olive green color) treatments; (b) volcano plot and GO keywords regarding the “Control + AMF vs Control”, “CM-LN1-L-SSH MNPs vs Control”, and “CM-LN1-L-SSH MNPs + AMF vs Control” comparisons; upregulated and downregulated pathways are highlighted in green and red, respectively.

Proteomic and gene ontology (GO) analyses have been performed to detect the differently represented proteins (DRP) and pathways involved in the magnetothermal treatment (Figure 11). The proteomic analyses were carried out for “Control”, “Control + AMF”, “CM-LN1-L-SSH MNPs”, and “CM-LN1-L-SSH MNPs + AMF” experimental classes.

The principal component analysis (PCA) of the first 2 components (accounting for 35.4% and 12.7% of the variance) is shown in Figure 11a (the symbols represent the four independent experiments for each experimental class). The component pattern plot of the PCA highlighted as “CM-LN1-L-SSH MNPs + AMF” represents the treatment with the most significant phenotypic variation compared to “Control”. This result demonstrates the synergic effect of the AMF stimulation and the treatment with CM-LN1-L-SSH MNPs. The “Control + AMF” and “CM-LN1-L-SSH MNPs” groups are positioned near the “Control” in the component pattern plot, demonstrating the safety of the single stimuli used for magnetic hyperthermia, in line with the previous results on cell viability, *K*_i_-67 expression, apoptosis/necrosis, hsp70 expression, and caspase-9 expression.

Figure 11b shows the volcano plots and the associated GO keywords of the 3 comparisons: “Control + AMF vs Control”, “CM-LN1-L-SSH MNPs vs Control”, and “CM-LN1-L-SSH MNPs + AMF vs Control”. The signaling pathway upregulation and downregulation compared to “Control” is, respectively, highlighted in green and red. In line with the results of the PCA, the comparison showing the higher number of DRP is “CM-LN1-L-SSH MNPs + AMF vs Control” (DRP = 2408 of 5908 detected proteins). The “CM-LN1-L-SSH MNPs vs Control” comparison showed a remarkably lower number of DRP (DRP = 127) compared to “CM-LN1-L-SSH MNPs + AMF vs Control”. “Control + AMF vs Control” comparisons did not show any DRP, highlighting the scarce interference that the magnetic field has at these frequencies and intensities. Since no DRP was found in “Control + AMF vs Control” comparison, no GO keywords have been highlighted for this comparison.

Significant GO terms involved in the “CM-LN1-L-SSH MNPs” treatment are “Cellular response to external stimulus”, “Negative regulation of autophagy”, “Extracellular matrix organization”, and “Extracellular encapsulating structure organization”. The upregulation of the “Cellular response to external stimulus” indicates that the PC-3 cells recognize the presence of the nanoparticles and activate a cellular response to this stimulus. Considering that a similar number of DRPs involved in the negative (DRP = 10) and positive (DRP = 12) regulation of the extrinsic apoptotic signaling pathways have been expressed in the “CM-LN1-L-SSH MNPs” group, the cell response to the external stimulus may not involve apoptosis. This result is in line with the apoptosis/necrosis evaluation. The “Negative regulation of autophagy” can be associated with the accumulation of nanomaterial and undesirable components in the cells.^88^ Instead, the “Extracellular structure organization”, “Extracellular matrix organization”, and “Extracellular encapsulating structure organization” are associated with the migration and invasion of cancer cells in the extracellular matrix and their upregulation,^89^ and they may explain the decreased migration levels in the “CM-LN1-L-SSH MNPs” treatment.

Downregulated GO terms in the “CM-LN1-L-SSH MNPs + AMF” group are “Cytoskeleton organization”, “Actin cytoskeleton organization”, “Cell cycle”, “Cell division”, and “Protein phosphorylation”, while upregulated GO terms include “Intracellular transport”. The downregulation of “Cytoskeleton organization” and of “Actin cytoskeleton organization” is associated with the decreased migration observed in response to the “CM-LN1-L-SSH MNPs + AMF” treatment. Considering that “Protein phosphorylation” is the most common mechanism of cell signaling regulation, its downregulation can be attributed to the impaired activity of dying cells, in the late phases of apoptosis and necrosis.^90^ The downregulation of “Cell cycle” and “Cell division” signaling pathways is a key requirement for the development of efficient anticancer treatment. Indeed, the scarce proliferation of the cancer cells that survived to the “CM-LN1-L-SSH MNPs + AMF” magnetothermal treatment may limit cancer aggressiveness and the probability of recurrence. The upregulation of the “Intracellular transport” is associated with elevated apoptotic levels and is due to the elevation of adenosine triphosphate (ATP) concentration in apoptotic cells.^91^ Indeed, intracellular transport is known to be accelerated during apoptosis (especially during the early phases): the increased intracellular transport is known to be critical for apoptotic mechanisms, and apoptosis can be delayed if it is regulated back to the normal level.^81^

The complete list of the GO terms related to the “CM-LN1-L-SSH MNPs” and “CM-LN1-L-SSH MNPs + AMF” groups has been reported as Supporting Information in Figure S13 and Figure S14, respectively (the number of genes, the fold enrichment, and the false discovery rate related to each GO term have been specified).

## 4 Conclusions

We reported on a novel class of magnetic core–shell–shell nanoparticles (i.e., trimagnetic nanoparticles) that consist of soft–soft–hard (SSH) nanostructure with enhanced heating ability (i.e., specific absorption rate) upon alternating magnetic field stimulation. We demonstrated that cell-membrane-coated and cell-penetrating peptide-conjugated nanoparticles improve the targeting of prostate cancer cells *in vitro*. We evidenced that the enhanced magnetothermal conversion efficiency of this system induced cell death mainly through the apoptosis process. The chronic magnetic hyperthermia treatment induced cell death through the caspase-9 pathway and significantly downregulated the cell cycle progression and cell-division-related pathways. Moreover, a decreased migration level has been observed in response to the magnetothermal treatment. Since the propensity of cancer cells to migrate correlates with tumor invasiveness,^92^ the proposed stimulation approach may have an important function *in vivo* in reducing the ability of tumor cells to invade surrounding tissues. The reduced migration in stimulated cells may be attributed to the significant downregulation of the cytoskeleton organization pathways observed with proteomics and GO analysis.

## Supporting Information Available

Fourteen figures, four tables, and an accurate description of XPS analysis (PDF)

## References

[ref1] Huo Y.; Yu J.; Gao S. Synthesis and Biomedical applications of Magnetic Nanomaterials. In Magnetic-mediated Hyperthermia for Cancer Treatment: Research Progress and Clinical Trials; EDP Sciences: Les Ulis, 2022; pp 228–260.

[ref2] Li B.; Chen X.; Qiu W.; Zhao R.; Duan J.; Zhang S.; Pan Z.; Zhao S.; Guo Q.; Qi Y.; Wang W.; Deng L.; Ni S.; Sang Y.; Xue H.; Liu H.; Li G. Synchronous Disintegration of Ferroptosis Defense Axis via Engineered Exosome-Conjugated Magnetic Nanoparticles for Glioblastoma Therapy. Adv. Sci. 2022, 9 (17), 2105451 10.1002/advs.202105451. PMC9189685 35508804

[ref3] Manohar A.; Vijayakanth V.; Vattikuti S. V. P.; Manivasagan P.; Jang E. S.; Chintagumpala K.; Kim K. H. Ca-Doped MgFe_2_O_4_ Nanoparticles for Magnetic Hyperthermia and Their Cytotoxicity in Normal and Cancer Cell Lines. ACS Appl. Nano Mater. 2022, 5 (4), 5847–5856. 10.1021/acsanm.2c01062.

[ref4] Kossatz S.; Grandke J.; Couleaud P.; Latorre A.; Aires A.; Crosbie-Staunton K.; Ludwig R.; Dähring H.; Ettelt V.; Lazaro-Carrillo A.; Calero M.; Sader M.; Courty J.; Volkov Y.; Prina-Mello A.; Villanueva A.; Somoza C.; Cortajarena A. L.; Miranda R.; Hilger I. Efficient Treatment of Breast Cancer Xenografts with Multifunctionalized Iron Oxide Nanoparticles Combining Magnetic Hyperthermia and Anti-Cancer Drug Delivery. Breast Cancer Res. 2015, 17 (1), 66 10.1186/s13058-015-0576-1. 25968050 PMC4451751

[ref5] Beola L.; Grazú V.; Fernández-Afonso Y.; Fratila R. M.; De Las Heras M.; De La Fuente J. M.; Gutiérrez L.; Asín L. Critical Parameters to Improve Pancreatic Cancer Treatment Using Magnetic Hyperthermia: Field Conditions, Immune Response, and Particle Biodistribution. ACS Appl. Mater. Interfaces 2021, 13 (11), 12982–12996. 10.1021/acsami.1c02338. 33709682 PMC8892434

[ref6] Eurostat Web Page. https://ec.europa.eu/eurostat/statistics-explained/index.php?title=Cancer_statistics_-_specific_cancers#Causes_of_death (accessed 2022-08-21).

[ref7] Chang D.; Lim M.; Goos J. A. C. M.; Qiao R.; Ng Y. Y.; Mansfeld F. M.; Jackson M.; Davis T. P.; Kavallaris M. Biologically Targeted Magnetic Hyperthermia: Potential and Limitations. Front. Pharmacol. 2018, 9, 831 10.3389/fphar.2018.00831. 30116191 PMC6083434

[ref8] Laha S. S.; Thorat N. D.; Singh G.; Sathish C. I.; Yi J.; Dixit A.; Vinu A. Rare-Earth Doped Iron Oxide Nanostructures for Cancer Theranostics: Magnetic Hyperthermia and Magnetic Resonance Imaging. Small 2022, 18 (11), 2104855 10.1002/smll.202104855. 34874618

[ref9] Das P.; Colombo M.; Prosperi D. Recent Advances in Magnetic Fluid Hyperthermia for Cancer Therapy. Colloids Surfaces B Biointerfaces 2019, 174, 42–55. 10.1016/j.colsurfb.2018.10.051. 30428431

[ref10] Caro C.; Egea-Benavente D.; Polvillo R.; Royo J. L.; Pernia Leal M.; García-Martín M. L. Comprehensive Toxicity Assessment of PEGylated Magnetic Nanoparticles for in Vivo Applications. Colloids Surfaces B Biointerfaces 2019, 177, 253–259. 10.1016/j.colsurfb.2019.01.051. 30763790

[ref11] Malhotra N.; Lee J.-S.; Liman R. A. D.; Ruallo J. M. S.; Villaflores O. B.; Ger T.-R.; Hsiao C.-D. Potential Toxicity of Iron Oxide Magnetic Nanoparticles: A Review. Molecules 2020, 25 (14), 3159 10.3390/molecules25143159. 32664325 PMC7397295

[ref12] Liu X.; Zhang Y.; Wang Y.; Zhu W.; Li G.; Ma X.; Zhang Y.; Chen S.; Tiwari S.; Shi K.; Zhang S.; Fan H. M.; Zhao Y. X.; Liang X. J. Comprehensive Understanding of Magnetic Hyperthermia for Improving Antitumor Therapeutic Efficacy. Theranostics 2020, 10 (8), 3793–3815. 10.7150/thno.40805. 32206123 PMC7069093

[ref13] Erofeev A.; Gorelkin P.; Garanina A.; Alova A.; Efremova M.; Vorobyeva N.; Edwards C.; Korchev Y.; Majouga A. Novel Method for Rapid Toxicity Screening of Magnetic Nanoparticles. Sci. Rep. 2018, 8 (1), 1–11. 10.1038/s41598-018-25852-4. 29748550 PMC5945642

[ref14] Setia A.; Mehata A. K.; Vikas; Malik A. K.; Viswanadh M. K.; Muthu M. S. Theranostic Magnetic Nanoparticles: Synthesis, Properties, Toxicity, and Emerging Trends for Biomedical Applications. J. Drug Delivery Sci. Technol. 2023, 81, 104295 10.1016/j.jddst.2023.104295.

[ref15] Liu X.; Zhang Y.; Wang Y.; Zhu W.; Li G.; Ma X.; Zhang Y.; Chen S.; Tiwari S.; Shi K.; Zhang S.; Fan H. M.; Zhao Y. X.; Liang X. J. Comprehensive Understanding of Magnetic Hyperthermia for Improving Antitumor Therapeutic Efficacy. Theranostics 2020, 10 (8), 3793–3815. 10.7150/thno.40805. 32206123 PMC7069093

[ref16] Walter A.; Billotey C.; Garofalo A.; Ulhaq-Bouillet C.; Lefevre C.; Taleb J.; Laurent S.; Vander Elst L.; Muller R. N.; Lartigue L.; Gazeau F.; Felder-Flesch D.; Begin-Colin S. Mastering the Shape and Composition of Dendronized Iron Oxide Nanoparticles to Tailor Magnetic Resonance Imaging and Hyperthermia. Chem. Mater. 2014, 26 (18), 5252–5264. 10.1021/cm5019025.

[ref17] Gavilán H.; Simeonidis K.; Myrovali E.; Mazarío E.; Chubykalo-Fesenko O.; Chantrell R.; Balcells L.; Angelakeris M.; Morales M. P.; Serantes D. How Size, Shape and Assembly of Magnetic Nanoparticles Give Rise to Different Hyperthermia Scenarios. Nanoscale 2021, 13 (37), 15631–15646. 10.1039/D1NR03484G. 34596185

[ref18] Lee J.-H.; Jang J.; Choi J.; Moon S. H.; Noh S.; Kim J.; Kim J.-G.; Kim I.-S.; Park K. I.; Cheon J. Exchange-Coupled Magnetic Nanoparticles for Efficient Heat Induction. Nat. Nanotechnol. 2011, 6 (7), 418–422. 10.1038/nnano.2011.95. 21706024

[ref19] Jun Y. W.; Seo J. W.; Cheon J. Nanoscaling Laws of Magnetic Nanoparticles and Their Applicabilities in Biomedical Sciences. Acc. Chem. Res. 2008, 41 (2), 179–189. 10.1021/ar700121f. 18281944

[ref20] Mai B. T.; Balakrishnan P. B.; Barthel M. J.; Piccardi F.; Niculaes D.; Marinaro F.; Fernandes S.; Curcio A.; Kakwere H.; Autret G.; Cingolani R.; Gazeau F.; Pellegrino T. Thermoresponsive Iron Oxide Nanocubes for an Effective Clinical Translation of Magnetic Hyperthermia and Heat-Mediated Chemotherapy. ACS Appl. Mater. Interfaces 2019, 11 (6), 5727–5739. 10.1021/acsami.8b16226. 30624889 PMC6376448

[ref21] Hammad M.; Nica V.; Hempelmann R. Synthesis and Characterization of Bi-Magnetic Core/Shell Nanoparticles for Hyperthermia Applications. IEEE Trans. Magn. 2017, 53 (4), 1 10.1109/TMAG.2016.2635696.

[ref22] Hammad M.; Nica V.; Hempelmann R. On-Off Switch-Controlled Doxorubicin Release from Thermo- and PH-Responsive Coated Bimagnetic Nanocarriers. J.Nanopart. Res. 2016, 18 (8), 234 10.1007/s11051-016-3550-7.

[ref23] Hammad M.; Nica V.; Hempelmann R. On-Command Controlled Drug Release by Diels-Alder Reaction Using Bi-Magnetic Core/Shell Nano-Carriers. Colloids Surfaces B Biointerfaces 2017, 150, 15–22. 10.1016/j.colsurfb.2016.11.005. 27865903

[ref24] Tapeinos C.; Marino A.; Battaglini M.; Migliorin S.; Brescia R.; Scarpellini A.; De Julián Fernández C.; Prato M.; Drago F.; Ciofani G. Stimuli-Responsive Lipid-Based Magnetic Nanovectors Increase Apoptosis in Glioblastoma Cells through Synergic Intracellular Hyperthermia and Chemotherapy. Nanoscale 2019, 11 (1), 72–88. 10.1039/C8NR05520C. PMC6336008 30357214

[ref25] Gavrilov-Isaac V.; Neveu S.; Dupuis V.; Taverna D.; Gloter A.; Cabuil V. Synthesis of Trimagnetic Multishell MnFe2O4@CoFe2O4@NiFe2O4 Nanoparticles. Small 2015, 11 (22), 2614–2618. 10.1002/smll.201402845. 25684735

[ref26] Nuñez J. M.; Hettler S.; Lima E.; Goya G. F.; Arenal R.; Zysler R. D.; Aguirre M. H.; Winkler E. L. Onion-like Fe3O4/MgO/CoFe2O4Magnetic Nanoparticles: New Ways to Control Magnetic Coupling between Soft and Hard Magnetic Phases. J. Mater. Chem. C 2022, 10 (41), 15339–15352. 10.1039/D2TC03144B.

[ref27] Grauer O.; Jaber M.; Hess K.; Weckesser M.; Schwindt W.; Maring S.; Wölfer J.; Stummer W. Combined Intracavitary Thermotherapy with Iron Oxide Nanoparticles and Radiotherapy as Local Treatment Modality in Recurrent Glioblastoma Patients. J. Neurooncol. 2019, 141 (1), 83–94. 10.1007/s11060-018-03005-x. 30506500 PMC6341053

[ref28] Park Y.; Demessie A. A.; Luo A.; Taratula O. R.; Moses A. S.; Do P.; Campos L.; Jahangiri Y.; Wyatt C. R.; Albarqi H. A.; Farsad K.; Slayden O. D.; Taratula O. Targeted Nanoparticles with High Heating Efficiency for the Treatment of Endometriosis with Systemically Delivered Magnetic Hyperthermia. Small 2022, 18 (24), 1–15. 10.1002/smll.202107808. PMC9232988 35434932

[ref29] Guntnur R. T.; Muzzio N.; Gomez A.; Macias S.; Galindo A.; Ponce A.; Romero G. On-Demand Chemomagnetic Modulation of Striatal Neurons Facilitated by Hybrid Magnetic Nanoparticles. Adv. Funct. Mater. 2022, 32, 2204732 10.1002/adfm.202204732. 36339020 PMC9635318

[ref30] Pucci C.; Degl’Innocenti A.; Belenli Gümüş M.; Ciofani G. Superparamagnetic Iron Oxide Nanoparticles for Magnetic Hyperthermia: Recent Advancements, Molecular Effects, and Future Directions in the Omics Era. Biomater. Sci. 2022, 10 (9), 2103–2121. 10.1039/D1BM01963E. 35316317

[ref31] Pudlarz A.; Szemraj J. Nanoparticles as Carriers of Proteins, Peptides and Other Therapeutic Molecules. Open Life Sci. 2018, 13 (1), 285–298. 10.1515/biol-2018-0035. 33817095 PMC7874720

[ref32] Longoria-García S.; Sánchez-Domínguez C. N.; Gallardo-Blanco H. Recent Applications of Cell-Penetrating Peptide Guidance of Nanosystems in Breast and Prostate Cancer (Review). Oncol. Lett. 2022, 23 (3), 103 10.3892/ol.2022.13223. 35154434 PMC8822396

[ref33] Zhu L.; Zhong Y.; Wu S.; Yan M.; Cao Y.; Mou N.; Wang G.; Sun D.; Wu W. Cell Membrane Camouflaged Biomimetic Nanoparticles: Focusing on Tumor Theranostics. Mater. Today Bio 2022, 14, 100228 10.1016/j.mtbio.2022.100228. PMC8898969 35265826

[ref34] Rao L.; Cai B.; Bu L. L.; Liao Q. Q.; Guo S. S.; Zhao X. Z.; Dong W. F.; Liu W. Microfluidic Electroporation-Facilitated Synthesis of Erythrocyte Membrane-Coated Magnetic Nanoparticles for Enhanced Imaging-Guided Cancer Therapy. ACS Nano 2017, 11 (4), 3496–3505. 10.1021/acsnano.7b00133. 28272874

[ref35] Yu G. T.; Rao L.; Wu H.; Yang L. L.; Bu L. L.; Deng W. W.; Wu L.; Nan X.; Zhang W. F.; Zhao X. Z.; Liu W.; Sun Z. J. Myeloid-Derived Suppressor Cell Membrane-Coated Magnetic Nanoparticles for Cancer Theranostics by Inducing Macrophage Polarization and Synergizing Immunogenic Cell Death. Adv. Funct. Mater. 2018, 28 (37), 1801389 10.1002/adfm.201801389.

[ref36] Kondo E.; Iioka H.; Saito K. Tumor-Homing Peptide and Its Utility for Advanced Cancer Medicine. Cancer Sci. 2021, 112 (6), 2118–2125. 10.1111/cas.14909. 33793015 PMC8177760

[ref37] Wada A.; Terashima T.; Kageyama S.; Yoshida T.; Narita M.; Kawauchi A.; Kojima H. Efficient Prostate Cancer Therapy with Tissue-Specific Homing Peptides Identified by Advanced Phage Display Technology. Mol. Ther. - Oncolytics 2019, 12 (3), 138–146. 10.1016/j.omto.2019.01.001. 30788426 PMC6369249

[ref38] Wang L.; Wang X.; Luo J.; Wanjala B. N.; Wang C.; Chernova N. A.; Engelhard M. H.; Liu Y.; Bae I. T.; Zhong C. J. Core-Shell-Structured Magnetic Ternary Nanocubes. J. Am. Chem. Soc. 2010, 132 (50), 17686–17689. 10.1021/ja1091084. 21121606

[ref39] Tong S.; Hou S.; Ren B.; Zheng Z.; Bao G. Self-Assembly of Phospholipid-PEG Coating on Nanoparticles through Dual Solvent Exchange. Nano Lett. 2011, 11 (9), 3720–3726. 10.1021/nl201978c. 21793503 PMC3173588

[ref40] De Pasquale D.; Marino A.; Tapeinos C.; Pucci C.; Rocchiccioli S.; Michelucci E.; Finamore F.; McDonnell L.; Scarpellini A.; Lauciello S.; Prato M.; Larrañaga A.; Drago F.; Ciofani G. Homotypic Targeting and Drug Delivery in Glioblastoma Cells through Cell Membrane-Coated Boron Nitride Nanotubes. Mater. Des. 2020, 192, 108742 10.1016/j.matdes.2020.108742. 32394995 PMC7212088

[ref41] Pucci C.; De Pasquale D.; Marino A.; Martinelli C.; Lauciello S.; Ciofani G. Hybrid Magnetic Nanovectors Promote Selective Glioblastoma Cell Death through a Combined Effect of Lysosomal Membrane Permeabilization and Chemotherapy. ACS Appl. Mater. Interfaces 2020, 12 (26), 29037–29055. 10.1021/acsami.0c05556. 32459082 PMC7343532

[ref42] Rasband W. S. ImageJ; US National Institutes of Health: Bethesda, Maryland, USA, 1997–2018. https://imagej.nih.gov/ij/.

[ref43] Fairley N.; Fernandez V.; Richard-Plouet M.; Guillot-Deudon C.; Walton J.; Smith E.; Flahaut D.; Greiner M.; Biesinger M.; Tougaard S.; Morgan D.; Baltrusaitis J. Systematic and Collaborative Approach to Problem Solving Using X-Ray Photoelectron Spectroscopy. Appl. Surf. Sci. Adv. 2021, 5 (March), 100112 10.1016/j.apsadv.2021.100112.

[ref44] https://www.malvernpanalytical.com/en/about-us/our-brands/panalytical.

[ref45] Fang C.; Veiseh O.; Kievit F.; Bhattarai N.; Wang F.; Stephen Z.; Li C.; Lee D.; Ellenbogen R. G.; Zhang M. Functionalization of Iron Oxide Magnetic Nanoparticles with Targeting Ligands: Their Physicochemical Properties and in Vivo Behavior. Nanomedicine 2010, 5 (9), 1357–1369. 10.2217/nnm.10.55. 21128719 PMC3057775

[ref46] Shi W.; Cao X.; Liu Q.; Zhu Q.; Liu K.; Deng T.; Yu Q.; Deng W.; Yu J.; Wang Q.; Xu X. Hybrid Membrane-Derived Nanoparticles for Isoliquiritin Enhanced Glioma Therapy. Pharmaceuticals 2022, 15 (9), 1059 10.3390/ph15091059. 36145280 PMC9506545

[ref47] Wildeboer R. R.; Southern P.; Pankhurst Q. A. On the Reliable Measurement of Specific Absorption Rates and Intrinsic Loss Parameters in Magnetic Hyperthermia Materials. J. Phys. D. Appl. Phys. 2014, 47 (49), 495003 10.1088/0022-3727/47/49/495003.

[ref48] Marino A.; Camponovo A.; Degl’Innocenti A.; Bartolucci M.; Tapeinos C.; Martinelli C.; De Pasquale D.; Santoro F.; Mollo V.; Arai S.; Suzuki M.; Harada Y.; Petretto A.; Ciofani G. Multifunctional Temozolomide-Loaded Lipid Superparamagnetic Nanovectors: Dual Targeting and Disintegration of Glioblastoma Spheroids by Synergic Chemotherapy and Hyperthermia Treatment. Nanoscale 2019, 11 (44), 21227–21248. 10.1039/C9NR07976A. 31663592 PMC6867905

[ref49] Avrutsky M. I.; Troy C. M. Caspase-9: A Multimodal Therapeutic Target with Diverse Cellular Expression in Human Disease. Front. Pharmacol. 2021, 12 (July), 1–17. 10.3389/fphar.2021.701301. PMC8299054 34305609

[ref50] Bruderer R.; Bernhardt O. M.; Gandhi T.; Miladinović S. M.; Cheng L. Y.; Messner S.; Ehrenberger T.; Zanotelli V.; Butscheid Y.; Escher C.; Vitek O.; Rinner O.; Reiter L. Extending the Limits of Quantitative Proteome Profiling with Data-Independent Acquisition and Application to Acetaminophen-Treated Three-Dimensional Liver Microtissues. Mol. Cell. Proteomics 2015, 14 (5), 1400–1410. 10.1074/mcp.M114.044305. 25724911 PMC4424408

[ref51] Tyanova S.; Temu T.; Sinitcyn P.; Carlson A.; Hein M. Y.; Geiger T.; Mann M.; Cox J. The Perseus Computational Platform for Comprehensive Analysis of (Prote)Omics Data. Nat. Methods 2016, 13 (9), 731–740. 10.1038/nmeth.3901. 27348712

[ref52] Nica V.; Sauer H. M.; Embs J.; Hempelmann R. Calorimetric Method for the Determination of Curie Temperatures of Magnetic Nanoparticles in Dispersion. J. Phys.: Condens. Matter 2008, 20 (20), 204115 10.1088/0953-8984/20/20/204115. 21694244

[ref53] Salazar-Alvarez G.; Lidbaum H.; López-Ortega A.; Estrader M.; Leifer K.; Sort J.; Suriñach S.; Baró M. D.; Nogués J. Two-, Three-, and Four-Component Magnetic Multilayer Onion Nanoparticles Based on Iron Oxides and Manganese Oxides. J. Am. Chem. Soc. 2011, 133 (42), 16738–16741. 10.1021/ja205810t. 21973012

[ref54] Grosvenor A. P.; Kobe B. A.; Biesinger M. C.; McIntyre N. S. Investigation of Multiplet Splitting of Fe 2p XPS Spectra and Bonding in Iron Compounds. Surf. Interface Anal. 2004, 36 (12), 1564–1574. 10.1002/sia.1984.

[ref55] Gupta R. P.; Sen S. K. Calculation of Multiplet Structure of Core p-Vacancy Levels. II. Phys. Rev. B 1975, 12 (1), 15–19. 10.1103/PhysRevB.12.15.

[ref56] McIntyre N. S.; Zetaruk D. G. X-Ray Photoelectron Spectroscopic Studies of Iron Oxides. Anal. Chem. 1977, 49 (11), 1521–1529. 10.1021/ac50019a016.

[ref57] Pratt A. R.; Muir I. J.; Nesbitt H. W. X-Ray Photoelectron and Auger Electron Spectroscopic Studies of Pyrrhotite and Mechanism of Air Oxidation. Geochim. Cosmochim. Acta 1994, 58 (2), 827–841. 10.1016/0016-7037(94)90508-8.

[ref58] Biesinger M. C.; Payne B. P.; Grosvenor A. P.; Lau L. W. M.; Gerson A. R.; Smart R. S. C. Resolving Surface Chemical States in XPS Analysis of First Row Transition Metals, Oxides and Hydroxides: Cr, Mn, Fe, Co and Ni. Appl. Surf. Sci. 2011, 257 (7), 2717–2730. 10.1016/j.apsusc.2010.10.051.

[ref59] Biesinger M. C.; Lau L. W. M.; Gerson A. R.; Smart R. S. C. Resolving Surface Chemical States in XPS Analysis of First Row Transition Metals, Oxides and Hydroxides: Sc, Ti, V, Cu and Zn. Appl. Surf. Sci. 2010, 257 (3), 887–898. 10.1016/j.apsusc.2010.07.086.

[ref60] Tanuma S.; Powell C. J.; Penn D. R. Electron Inelastic Mean Free Paths in Solids at Low Energies. J. Electron Spectrosc. Relat. Phenom. 1990, 52 (C), 285–291. 10.1016/0368-2048(90)85024-4.

[ref61] Song Q.; Zhang Z. J. Controlled Synthesis and Magnetic Properties of Bimagnetic Spinel Ferrite CoFe2O4 and MnFe2O 4 Nanocrystals with Core-Shell Architecture. J. Am. Chem. Soc. 2012, 134 (24), 10182–10190. 10.1021/ja302856z. 22621435

[ref62] Juhin A.; López-Ortega A.; Sikora M.; Carvallo C.; Estrader M.; Estradé S.; Peiró F.; Baró M. D.; Sainctavit P.; Glatzel P.; Nogués J. Direct Evidence for an Interdiffused Intermediate Layer in Bi-Magnetic Core-Shell Nanoparticles. Nanoscale 2014, 6 (20), 11911–11920. 10.1039/C4NR02886D. 25174899

[ref63] Kubisztal M.; Kubisztal J.; Karolus M.; Prusik K.; Haneczok G. Evolution of Frozen Magnetic State in Co-Precipitated ZnδCo_1_-δFe_2_O_4_ (0 ≤ δ ≤ 1) Ferrite Nanopowders. J. Magn. Magn. Mater. 2018, 454, 368–374. 10.1016/j.jmmm.2018.02.001.

[ref64] Gneveckow U.; Jordan A.; Scholz R.; Brüß V.; Waldöfner N.; Ricke J.; Feussner A.; Hildebrandt B.; Rau B.; Wust P. Description and Characterization of the Novel Hyperthermia- and Thermoablation-System MFH®300F for Clinical Magnetic Fluid Hyperthermia. Med. Phys. 2004, 31 (6), 1444–1451. 10.1118/1.1748629. 15259647

[ref65] Liu X.; Zhang Y.; Wang Y.; Zhu W.; Li G.; Ma X.; Zhang Y.; Chen S.; Tiwari S.; Shi K.; Zhang S.; Fan H. M.; Zhao Y. X.; Liang X. J. Comprehensive Understanding of Magnetic Hyperthermia for Improving Antitumor Therapeutic Efficacy. Theranostics 2020, 10 (8), 3793–3815. 10.7150/thno.40805. 32206123 PMC7069093

[ref66] Noh S. H.; Na W.; Jang J. T.; Lee J. H.; Lee E. J.; Moon S. H.; Lim Y.; Shin J. S.; Cheon J. Nanoscale Magnetism Control via Surface and Exchange Anisotropy for Optimized Ferrimagnetic Hysteresis. Nano Lett. 2012, 12 (7), 3716–3721. 10.1021/nl301499u. 22720795

[ref67] Balk M.; Haus T.; Band J.; Unterweger H.; Schreiber E.; Friedrich R. P.; Alexiou C.; Gostian A. O. Cellular Spion Uptake and Toxicity in Various Head and Neck Cancer Cell Lines. Nanomaterials 2021, 11 (3), 726 10.3390/nano11030726. 33805818 PMC7999062

[ref68] Chen L.; Hong W.; Ren W.; Xu T.; Qian Z.; He Z. Recent Progress in Targeted Delivery Vectors Based on Biomimetic Nanoparticles. Signal Transduct. Target. Ther. 2021, 6, 225 10.1038/s41392-021-00631-2. 34099630 PMC8182741

[ref69] Li J.; Wang X.; Zheng D.; Lin X.; Wei Z.; Zhang D.; Li Z.; Zhang Y.; Wu M.; Liu X. Cancer Cell Membrane-Coated Magnetic Nanoparticles for MR/NIR Fluorescence Dual-Modal Imaging and Photodynamic Therapy. Biomater. Sci. 2018, 6 (7), 1834–1845. 10.1039/C8BM00343B. 29786715

[ref70] Weiss I. M.; Muth C.; Drumm R.; Kirchner H. O. K. Thermal Decomposition of the Amino Acids Glycine, Cysteine, Aspartic Acid, Asparagine, Glutamic Acid, Glutamine, Arginine and Histidine. BMC Biophys. 2018, 11, 2 10.1186/s13628-018-0042-4. 29449937 PMC5807855

[ref71] Athanasoulia I. G.; Tarantili P. A. Preparation and Characterization of Polyethylene Glycol/Poly(L-Lactic Acid) Blends. Pure Appl. Chem. 2017, 89 (1), 141–152. 10.1515/pac-2016-0919.

[ref72] Xia X.; Yang M.; Wang Y.; Zheng Y.; Li Q.; Chen J.; Xia Y. Quantifying the Coverage Density of Poly(Ethylene Glycol) Chains on the Surface of Gold Nanostructures. ACS Nano 2012, 6 (1), 512–522. 10.1021/nn2038516. 22148912 PMC3265621

[ref73] Miller I.; Min M.; Yang C.; Tian C.; Gookin S.; Carter D.; Spencer S. L. Ki67 Is a Graded Rather than a Binary Marker of Proliferation versus Quiescence. Cell Rep. 2018, 24 (5), 1105–1112. 10.1016/j.celrep.2018.06.110. 30067968 PMC6108547

[ref74] Nguyen T. N.; Chebbi I.; Le Fèvre R.; Guyot F.; Alphandéry E. Non-Pyrogenic Highly Pure Magnetosomes for Efficient Hyperthermia Treatment of Prostate Cancer. Appl. Microbiol. Biotechnol. 2023, 107 (4), 1159–1176. 10.1007/s00253-022-12247-9. 36633624

[ref75] Calatayud M. P.; Soler E.; Torres T. E.; Campos-Gonzalez E.; Junquera C.; Ibarra M. R.; Goya G. F. Cell Damage Produced by Magnetic Fluid Hyperthermia on Microglial BV2 Cells. Sci. Rep. 2017, 7 (1), 1–16. 10.1038/s41598-017-09059-7. 28819156 PMC5561037

[ref76] Crezee J.; Franken N. A. P.; Oei A. L. Hyperthermia-Based Anti-Cancer Treatments. Cancers 2021, 13, 1240 10.3390/cancers13061240. 33808948 PMC7999567

[ref77] Sabirzhanov B.; Stoica B. A.; Hanscom M.; Piao C. S.; Faden A. I. Over-Expression of HSP70 Attenuates Caspase-Dependent and Caspase-Independent Pathways and Inhibits Neuronal Apoptosis. J. Neurochem. 2012, 123 (4), 542–554. 10.1111/j.1471-4159.2012.07927.x. 22909049 PMC3753080

[ref78] Calderwood S. K.; Gong J. Heat Shock Proteins Promote Cancer: It’s a Protection Racket. Trends Biochem. Sci. 2016, 41 (4), 311–323. 10.1016/j.tibs.2016.01.003. 26874923 PMC4911230

[ref79] Moulin M.; Arrigo A. P. Caspases Activation in Hyperthermia-Induced Stimulation of TRAIL Apoptosis. Cell Stress Chaperones 2008, 13 (3), 313–326. 10.1007/s12192-008-0027-3. 18330721 PMC2673937

[ref80] Court K. A.; Hatakeyama H.; Wu S. Y.; Lingegowda M. S.; Rodríguez-Aguayo C.; López-Berestein G.; Ju-Seog L.; Rinaldi C.; Juan E. J.; Sood A. K.; Torres-Lugo M. HSP70 Inhibition Synergistically Enhances the Effects of Magnetic Fluid Hyperthermia in Ovarian Cancer. Mol. Cancer Ther. 2017, 16 (5), 966–976. 10.1158/1535-7163.MCT-16-0519. 28223424

[ref81] Şen D.; Emanet M.; Ciofani G. Nanotechnology-Based Strategies to Evaluate and Counteract Cancer Metastasis and Neoangiogenesis. Adv. Healthc. Mater. 2021, 10 (10), 1–30. 10.1002/adhm.202002163. PMC7610913 33763992

[ref82] Soenen S. J. H.; Himmelreich U.; Nuytten N.; De Cuyper M. Cytotoxic Effects of Iron Oxide Nanoparticles and Implications for Safety in Cell Labelling. Biomaterials 2011, 32 (1), 195–205. 10.1016/j.biomaterials.2010.08.075. 20863560

[ref83] Mulens-Arias V.; Rojas J. M.; Sanz-ortega L.; Portilla Y.; Pérez-yagüe S.; Barber D. F. Polyethylenimine-Coated Superparamagnetic Iron Oxide Nanoparticles Impair *in vitro* and *in vivo* Angiogenesis ☆,☆☆,☆☆☆. Nanomedicine Nanotechnology, Biol. Med. 2019, 21, 102063 10.1016/j.nano.2019.102063. 31326525

[ref84] Wu V. M.; Huynh E.; Tang S.; Uskoković V. Brain and Bone Cancer Targeting by a Ferrofluid Composed of Superparamagnetic Iron-Oxide/Silica/Carbon Nanoparticles (Earthicles). Acta Biomater. 2019, 88, 422–447. 10.1016/j.actbio.2019.01.064. 30711662

[ref85] Hildebrandt B.; Wust P.; Ahlers O.; Dieing A.; Sreenivasa G.; Kerner T.; Felix R.; Riess H. The Cellular and Molecular Basis of Hyperthermia. Crit. Rev. Oncol. Hematol. 2002, 43 (1), 33–56. 10.1016/S1040-8428(01)00179-2. 12098606

[ref86] Jordan A.; Scholz R.; Maier-Hauff K.; Johannsen M.; Wust P.; Nadobny J.; Schirra H.; Schmidt H.; Deger S.; Loening S.; Lanksch W.; Felix R. Presentation of a New Magnetic Field Therapy System for the Treatment of Human Solid Tumors with Magnetic Fluid Hyperthermia. J. Magn. Magn. Mater. 2001, 225 (1–2), 118–126. 10.1016/S0304-8853(00)01239-7.

[ref87] Shi F.; Ma M.; Zhai R.; Ren Y.; Li K.; Wang H.; Xu C.; Huang X.; Wang N.; Zhou F.; Yao W. Overexpression of Heat Shock Protein 70 Inhibits Epithelial-Mesenchymal Transition and Cell Migration Induced by Transforming Growth Factor-β in A549 Cells. Cell Stress Chaperones 2021, 26 (3), 505–513. 10.1007/s12192-021-01196-3. 33598875 PMC8065086

[ref88] Liang C. Negative Regulation of Autophagy. Cell Death Differ. 2010, 17 (12), 1807–1815. 10.1038/cdd.2010.115. 20865012 PMC3131090

[ref89] Winkler J.; Abisoye-Ogunniyan A.; Metcalf K. J.; Werb Z. Concepts of Extracellular Matrix Remodelling in Tumour Progression and Metastasis. Nat. Commun. 2020, 11 (1), 1–19. 10.1038/s41467-020-18794-x. 33037194 PMC7547708

[ref90] Day E. K.; Sosale N. G.; Lazzara M. J. Cell Signaling Regulation by Protein Phosphorylation: A Multivariate, Heterogeneous, and Context-Dependent Process. Curr. Opin. Biotechnol. 2016, 40, 185–192. 10.1016/j.copbio.2016.06.005. 27393828 PMC4975652

[ref91] Li B.; Dou S. X.; Yuan J. W.; Liu Y. R.; Li W.; Ye F.; Wang P. Y.; Li H. Intracellular Transport Is Accelerated in Early Apoptotic Cells. Proc. Natl. Acad. Sci. U. S. A. 2018, 115 (48), 12118–12123. 10.1073/pnas.1810017115. 30429318 PMC6275518

[ref92] West A. K. V.; Wullkopf L.; Christensen A.; Leijnse N.; Tarp J. M.; Mathiesen J.; Erler J. T.; Oddershede L. B. Dynamics of Cancerous Tissue Correlates with Invasiveness. Sci. Rep. 2017, 7, 1–11. 10.1038/srep43800. 28262796 PMC5338316

