# Supplementary material for: Cell-Membrane-Coated and Cell-Penetrating Peptide-Conjugated Trimagnetic Nanoparticles for Targeted Magnetic Hyperthermia of Prostate Cancer Cells
Source: ACS Appl Mater Interfaces. 2023 Jun 13;15(25):30008–28. doi: 10.1021/acsami.3c07248 (PMC10316402; doi:10.1021/acsami.3c07248)
Supplement: Supplementary file 1 — am3c07248_si_001.pdf [file am3c07248_si_001.pdf]

Supporting Information

Cell Membrane-Coated and Cell-Penetrating Peptide-Conjugated Trimagnetic Nanoparticles for Targeted Magnetic Hyperthermia of Prostate Cancer Cells

*Valentin Nica[1, *], Attilio Marino[1, *] Carlotta Pucci[1], Özlem Şen[1], Melis Emanet[1], Daniele De Pasquale[1], Alessio Carmignani[1,2], Andrea Petretto[3], Martina Bartolucci[3], Simone Lauciello[4], Rosaria Brescia[4], Francesco de Boni[5], Mirko Prato[5], Sergio Marras[5], Filippo Drago[4], Mohaned Hammad[6], Doris Segets[6], Gianni Ciofani[1, *]*

[1]Istituto Italiano di Tecnologia, Smart Bio-Interfaces, Viale Rinaldo Piaggio 34, 56025 Pontedera, Italy

[2]Sant'Anna School of Advanced Studies, The Biorobotics Institute, Viale Rinaldo Piaggio 34, 56025 Pontedera, Italy

[3]IRCCS Istituto Giannina Gaslini, Core Facilities-Clinical Proteomics and Metabolomics, Via Gerolamo Gaslini 5, 16147 Genova, Italy

[4]Istituto Italiano di Tecnologia, Electron Microscopy Facility, Via Morego 30, 16163 Genova, Italy

[5]Istituto Italiano di Tecnologia, Materials Characterization Facility, Via Morego 30, 16163 Genova, Italy

[6]University of Duisburg-Essen, Particle Science and Technology - Institute for Combustion and Gas Dynamics (IVG-PST), Carl-Benz Strasse 199, 47057 Duisburg, Germany

*Corresponding authors: valentin.nica@iit.it, attilio.marino@iit.it, gianni.ciofani@iit.it

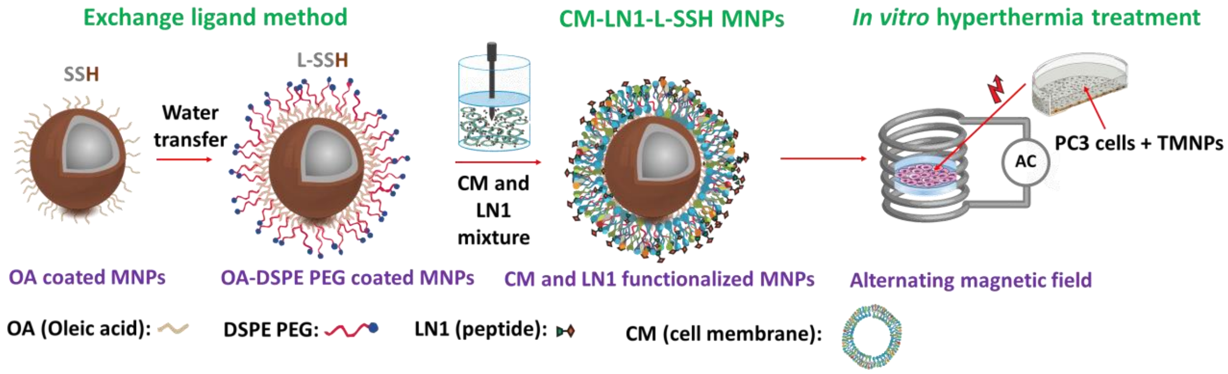

Figure S1. Schematic illustration of CM-LN1-L-SSH MNPs preparation and their application for *in vitro* prostate cancer treatment. $Fe_3O_4@Mn_{0.5}Zn_{0.5}Fe_2O_4@CoFe_2O_4$, soft-soft-hard (SSH).

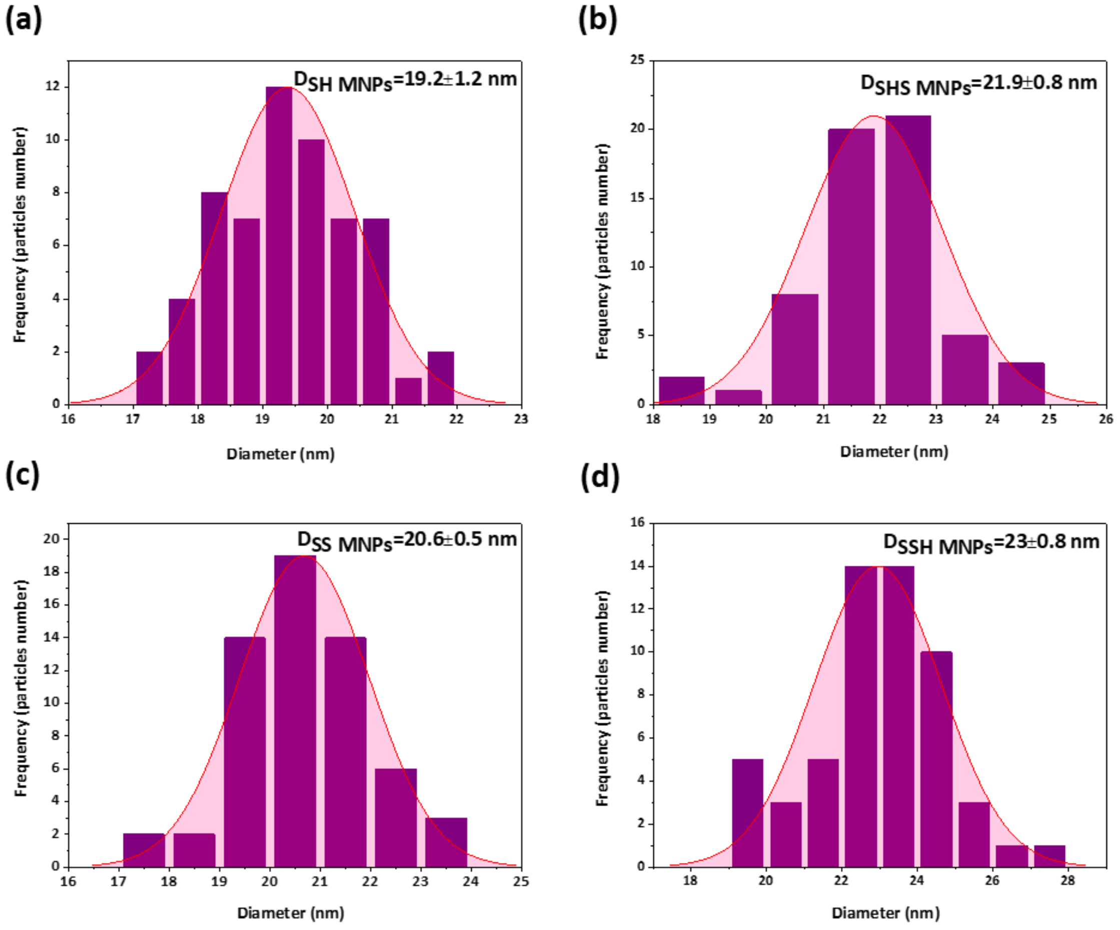

Figure S2. Size distribution histograms of (a) SH, (b) SHS, (c) SS and, (d) SSH MNPs.

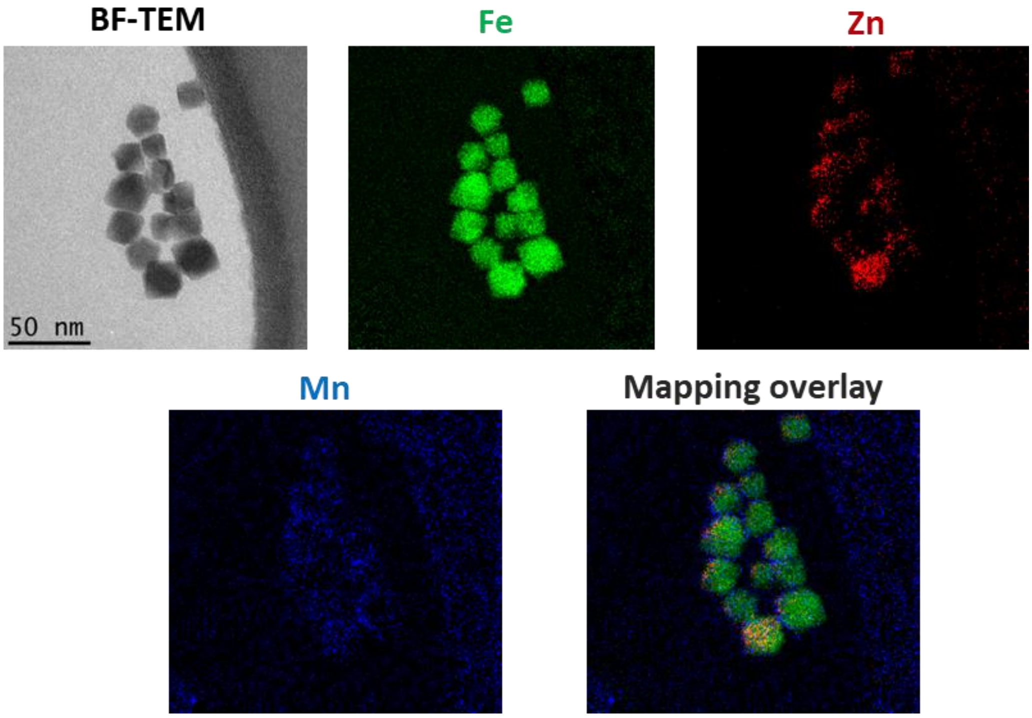

Figure S3. BF-TEM image and EFTEM elemental mapping for SS MNPs. EFTEM mapping demonstrate the presence of Fe, Zn, and Mn.

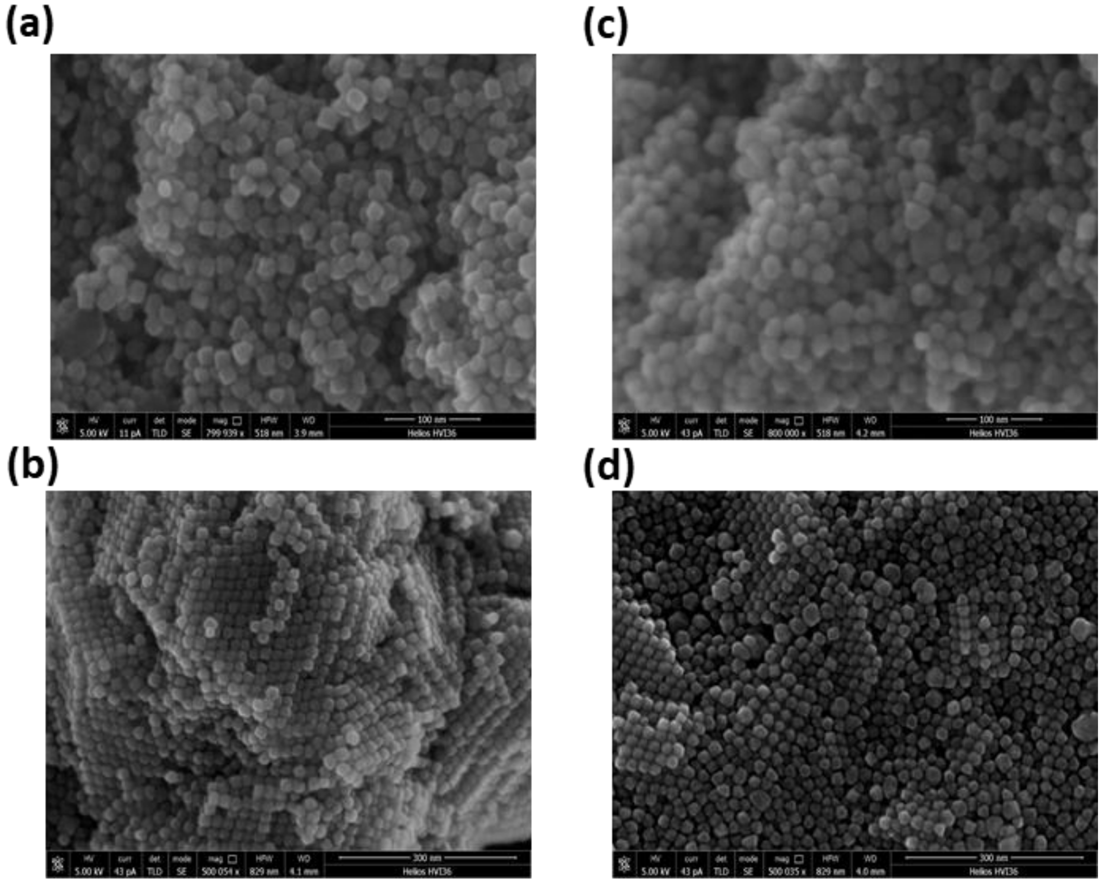

Figure S4. Representative SEM images of (a) SH, (b) SHS, (c) SS, and (d) SSH MNPs.

XPS Analysis

Figures S5 and S6 show, respectively, the wide scan and the high-resolution XPS spectra collected on SH, SS, SHS, and SSH MNPs. The high-resolution spectra are shown together with the results of the best fitting procedure.

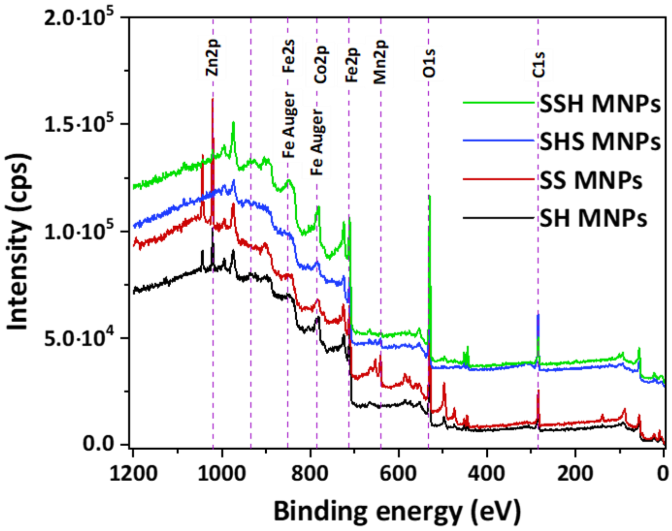

Figure S5. Wide XPS spectra of pristine SS, SH, SHS, and SSH MNPs.

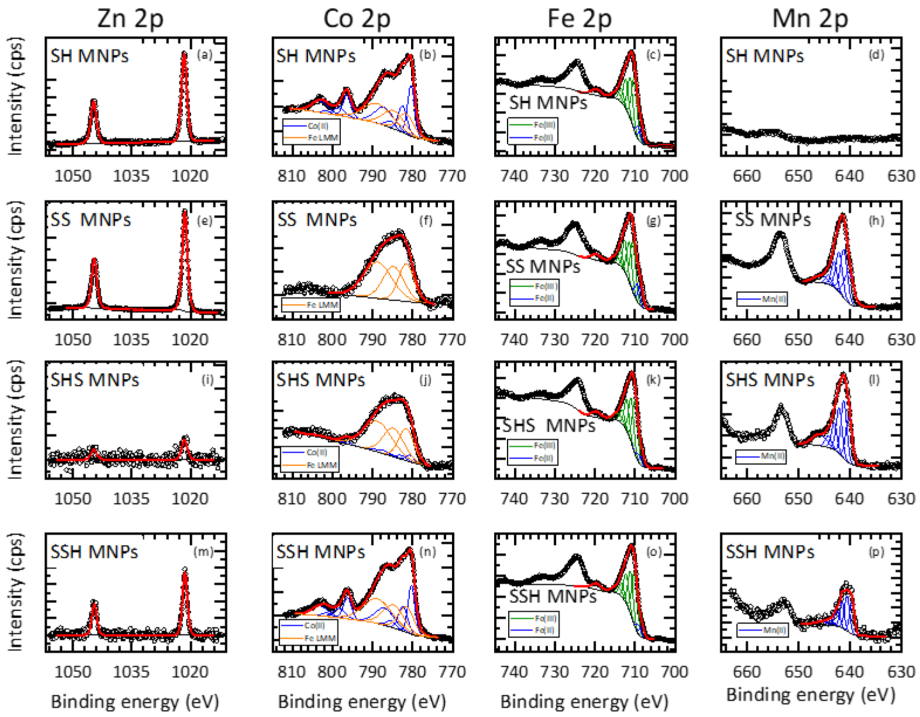

Figure S6. High-resolution XPS spectra of pristine samples (from top to bottom: SH, SS, SHS, and SSH MNPs) and corresponding elemental peak deconvolution of Zn 2p, Co 2p, Fe 2p, Mn 2p (from left to right). The background was subtracted to the Zn 2p region of samples SHS and SSH for a better comparison with the same regions of the other two samples. The small feature present in the Mn 2p region of sample SH is due to indium substrate (namely $In3p_{3/2}$ peak), as discussed in the text.

As reported in the main text, fitting of Co 2p, Fe 2p, and Mn 2p data is not straightforward, as multiplet splitting effects must be taken into account, together with usual spin-orbit coupling, shake-up and plasmon loss features. In the present case, moreover, fitting is complicated also by the overlap of different transitions in the same spectral region.

In this work, three overlaps were evaluated. The first one between the $Mn2p_{1/2}$ peak and a transition owing to the indium substrate, namely $In3p_{3/2}$. This feature is clearly visible in the spectrum of sample SH, which does not contain Mn (Figure S5). The problem was solved focusing only on $Mn2p_{3/2}$ peak in the analysis of Mn2p region: this is possible because the $Mn2p_{1/2}$ transition is well-separated from the $Mn2p_{3/2}$ one.

The second overlap is between the Co2p peak and a LMM Auger transition of Fe. This spectral feature was deconvoluted to avoid overestimating the relative amount of Co in the samples, according to the following procedure. First, this region was fitted for the SS sample, which does not contain Co, so that the only contribution in the 770 - 810 eV range is related to the Fe Auger peak. The best fit was achieved using three components, which have no specific chemical or physical meaning, but help to reproduce the experimental trend due to the Fe LMM transition. Then, these components were included in the fitting of the other three samples, which contain both Co and Fe. All the parameters determining the shape and the relative intensities of these components were fixed to the values obtained from the previous fit, letting only the overall intensity to change together with the peaks of Co2p, to reach the best fit of the whole region. In this way, the contribution of Fe Auger transition can be separated from the Co2p peak.

Finally, the last overlap that should be considered is between the Fe2p region and an LMM Auger transition of Co. However, the contribution of Co Auger peak was neglected for two reasons: 1) there are no samples containing Co but not Fe, so the previous procedure cannot be applied in this case, and 2) the amount of Co is much lower than the one of Fe, thus the overestimation of the Fe relative amount is expected to be limited.

Fitting of Mn 2p peaks

The broad and asymmetric peak around 641 eV corresponds to the $Mn2p_{3/2}$ photoemission in the case of SHS, SS, and SSH samples, while for the SH sample no Mn features are present and only a signal due to the indium substrate is detected, as discussed above. According to the literature[1], the $Mn2p_{3/2}$ photoemission peak is decomposed in six contributions centered at

(1) Biesinger, M. C.; Payne, B. P.; Grosvenor, A. P.; Lau, L. W. M.; Gerson, A. R.; Smart, R. S. C. Resolving Surface Chemical States in XPS Analysis of First Row Transition Metals, Oxides and Hydroxides: Cr, Mn, Fe, Co and Ni. *Appl. Surf. Sci.* **2011**, *257* (7), 2717–2730. https://doi.org/10.1016/j.apsusc.2010.10.051.

640.2, 641.1, 642.1, 643.0, 644.2 and 645.6 eV respectively, that are attributed to multiplet splitting of Mn in the 2+ oxidation state.

Fitting of Fe 2p peaks

For each sample, the XPS spectrum of Fe2p displays two main asymmetric peaks, $Fe2p_{3/2}$ and $Fe2p_{1/2}$, centered at 711.0 eV and 724.5 eV, respectively (Figure S5). These contributions are mainly due to Fe^{3+} cations. Moreover, a small shoulder centered at about 708.5 eV can be associated with the presence of Fe^{2+} species. Considering that both Fe(III) and Fe(II) signals undergo multiplet splitting, the $Fe2p_{3/2}$ peak was fitted with eight components: 710.2, 711.2, 712.3, 713.4, 714.5, and 719.6 eV owing to multiplet splitting of Fe^{3+}, and 708.4 and 709.2 eV due to multiplet splitting of Fe^{2+}. Also in this case, the parameters used in the fitting are based on the work of Biesinger *et al*[1]. The presence of Fe species with both 2+ and 3+ oxidation states can be explained considering the structure of these samples, as reported in the main text.

Fitting of Co 2p peaks

In the case of SH, SHS, and SSH samples, the Co2p spectrum has been fitted considering both $Co2p_{3/2}$ and $Co2p_{1/2}$ contribution (Figure S5), while the corresponding spectrum of SS sample, which does not contain Co, was used to evaluate the contribution of Fe Auger peak, as described above. In the Co2p spectrum of samples SH and SSH, the features with binding energy positioned at about 780.6 and 786.5 eV were attributed to $Co2p_{3/2}$ and its satellite. Accordingly, the components centered at 796.3 and 802.8 eV correspond to the $Co2p_{1/2}$ core level and its satellite, respectively. In the Co2p spectrum of the SHS sample these peaks are not clearly distinguishable because the Fe Auger component is predominant. Due to multiplet splitting, Co fitting was achieved *via* deconvolution of each $Co2p_{3/2}$ and $Co2p_{1/2}$ core-level photoemission peak into four components: 780.4, 782.5, 785.5, 787.1 eV, and 796.4, 798.3, 801.2, 803.2 eV, respectively. These components correspond to the presence of Co(II) in the samples[1].

Fitting of Zn 2p peaks

Differently from the other elements, Zn does not undergo multiplet splitting[2]. The presence in all the samples of core-level peaks at 1021.4 ($Zn2p_{3/2}$) and at 1044 eV ($Zn2p_{1/2}$) demonstrate the Zn^{2+} oxidation state.

(2) Biesinger, M. C.; Lau, L. W. M.; Gerson, A. R.; Smart, R. S. C. Resolving Surface Chemical States in XPS Analysis of First Row Transition Metals, Oxides and Hydroxides: Sc, Ti, V, Cu and Zn. *Appl. Surf. Sci.* **2010**, *257* (3), 887–898. https://doi.org/10.1016/j.apsusc.2010.07.086.

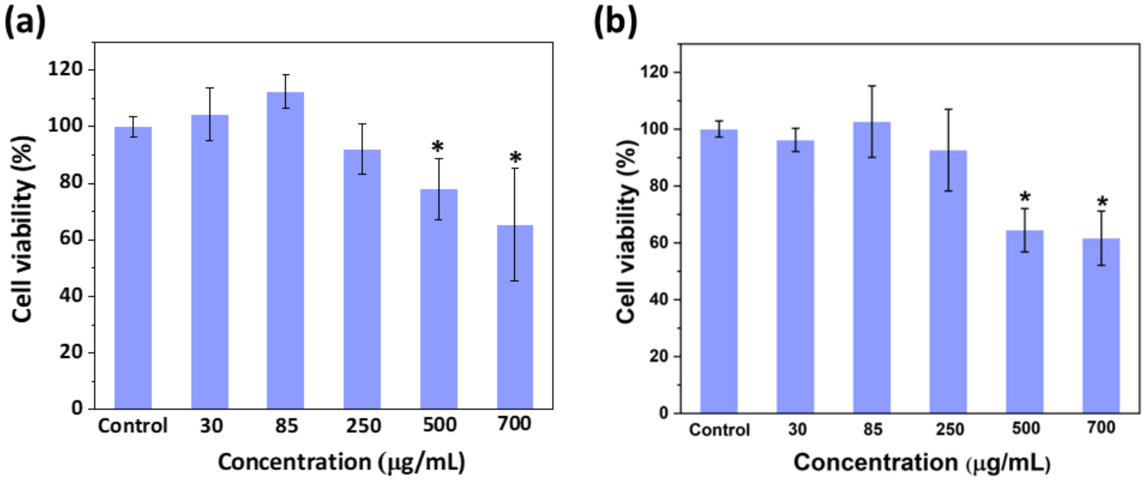

Figure S7. PC-3 cell viability at 72 h of treatment with different concentrations of L-SSH MNPs. For (a) PC3 cells and, (b) human primary normal prostate cells. All the values were normalized at the untreated control value; * $p < 0.05$.

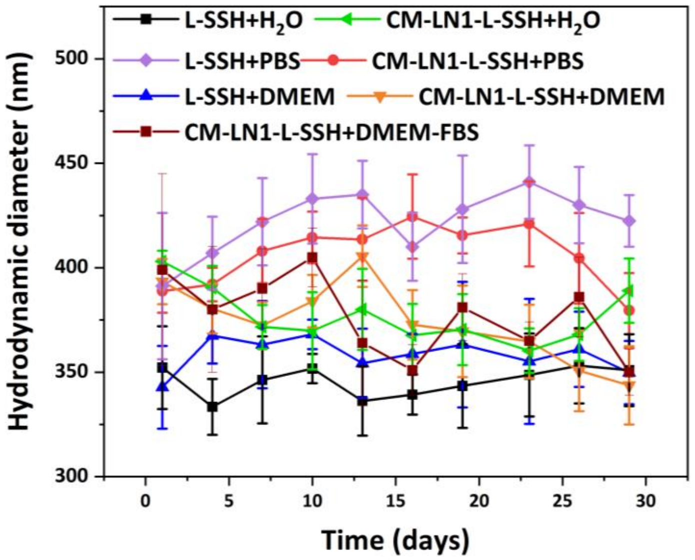

Figure S8. Stability of L-SSH in Milli-Q, PBS and DMEM, and the stability of CM-LN1-L-SSH MNPs in Milli-Q, PBS, DMEM, and 90% DMEM+10% FBS over 1 month.

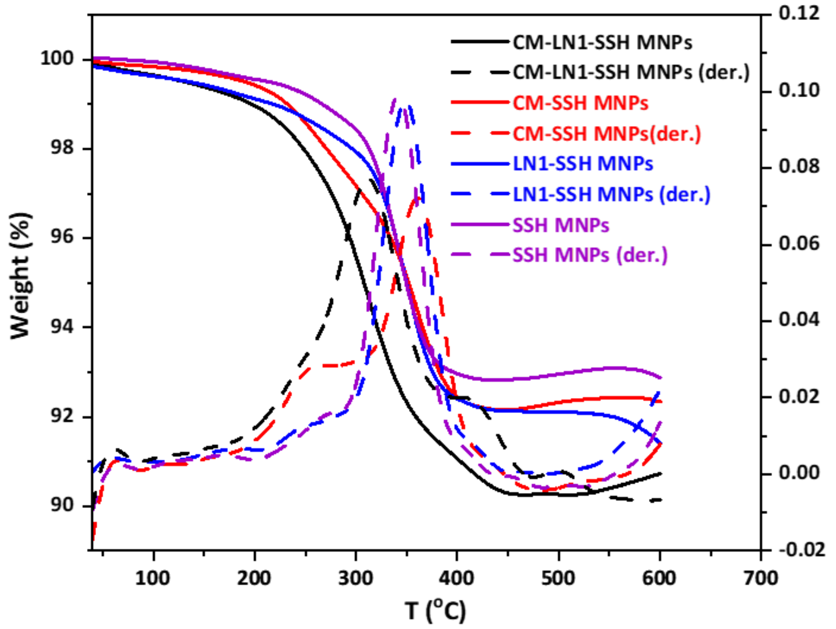

Figure S9. TGA showing the percentages of weight loss (solid line) and their corresponding derivative (dashed line) over the temperature for L-SSH, CM-L-SSH, LN1-L-SSH and CM-LN1-L-SSH samples.

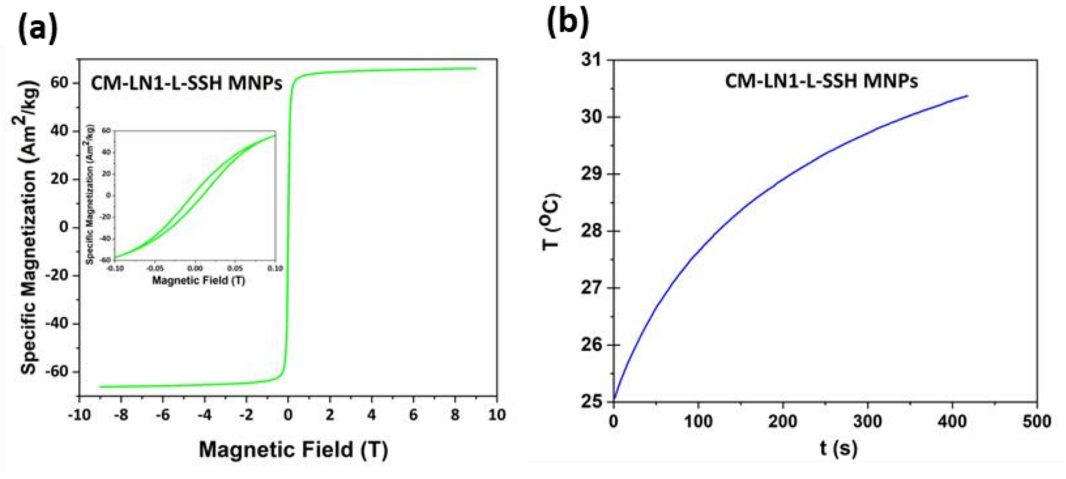

Figure S10. (a) Magnetic curve at room temperature of CM-LN1-L-SSH MNPs. The inset evidences the coercivity value of the sample. (b) Heating profile of CM-LN1-L-SSH MNPs exposed to AMF (f = 97.5 kHz, B = 20 mT, H = 15.9 kA/m; MNPs 5 mg/mL).

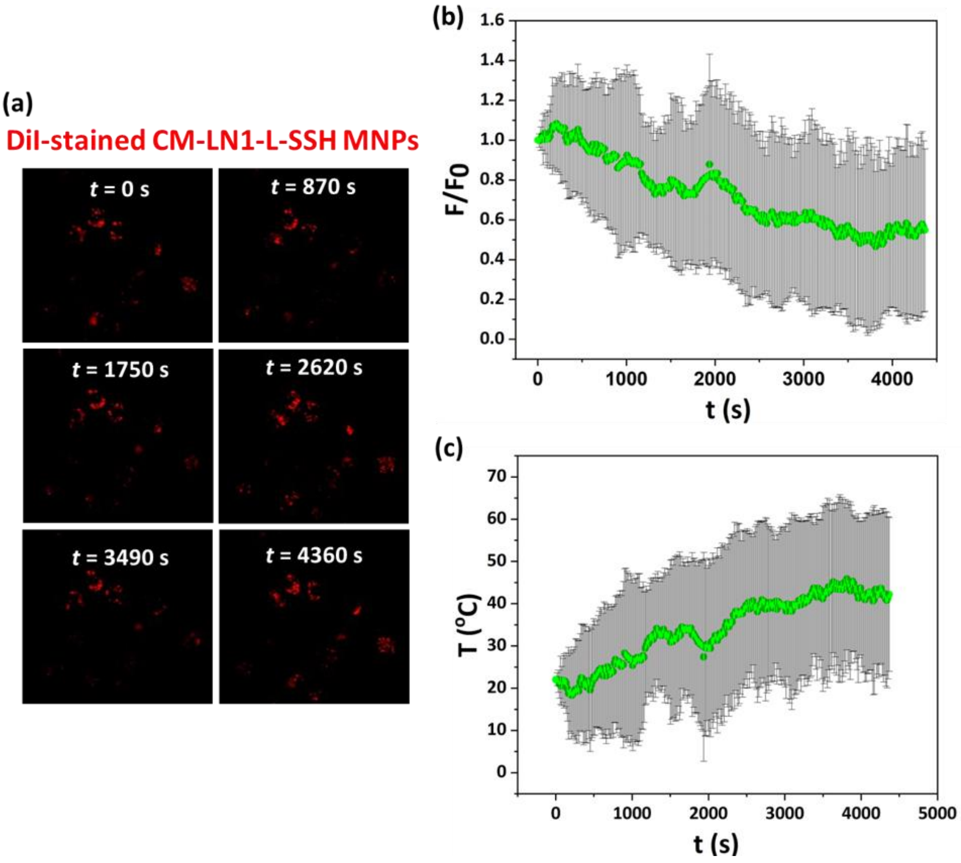

Figure S11. (a) Confocal laser scanning microscopy imaging of DiI-stained CM-LN1-L-SSH MNPs in cells upon magnetothermal stimulation; (b) fluorescence intensity of the CM-LN1-L-SSH MNPs *versus* time during AMF stimulation; (c) temperature of the CM-LN1-L-SSH MNPs *versus* time during AMF stimulation.

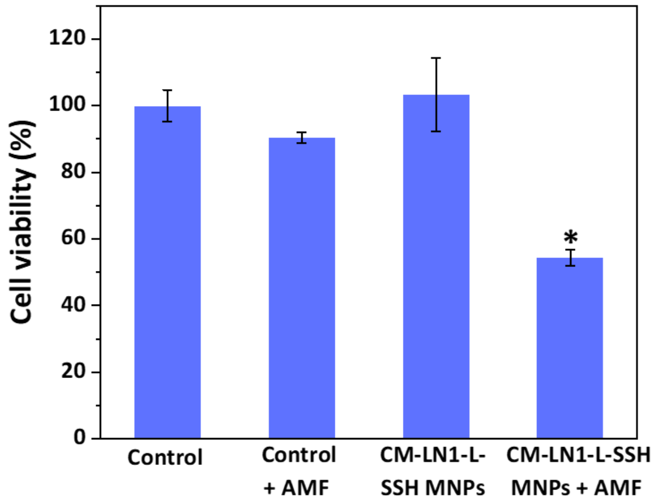

Figure S12. Cell viability of PC-3 cells in different experimental conditions: non-stimulated and non-treated controls (Control), cells stimulated with AMF (Control + AMF), cells treated with nanoparticles (CM-LN1-L-SSH MNPs), and cells stimulated with magnetic hyperthermia procedure (CM-LN1-L-SSH MNPs + AMF). * $p < 0.05$.

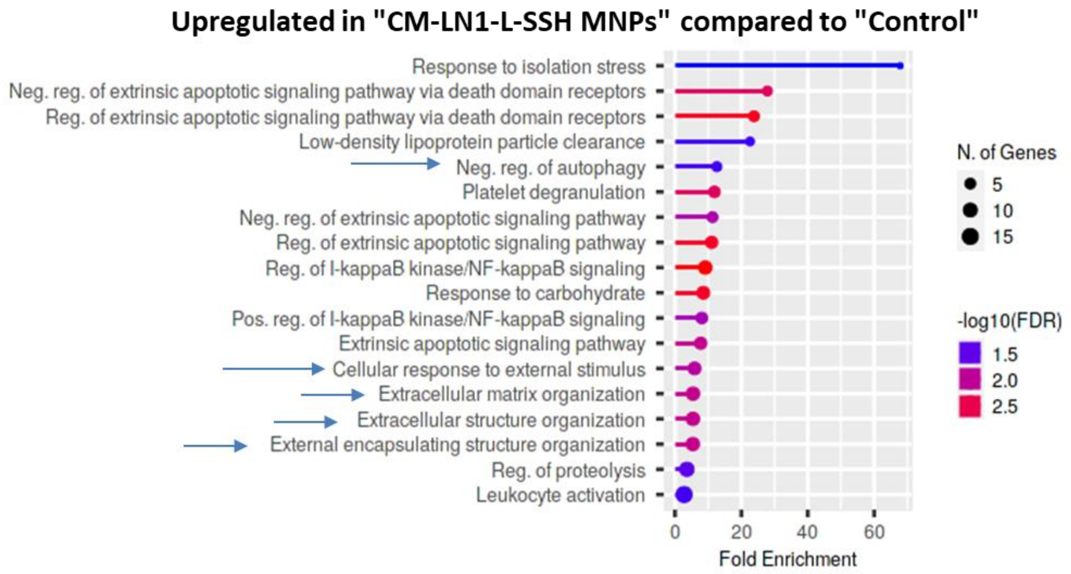

Figure S13. Proteomic analysis: complete list of the GO terms related to the “CM-LN1-L-SSH MNPs” experimental class. No downregulated GO term was found in “CM-LN1-L-SSH MNPs” compared to “Control”. Arrows indicate the regulated GO terms discussed in the main text.

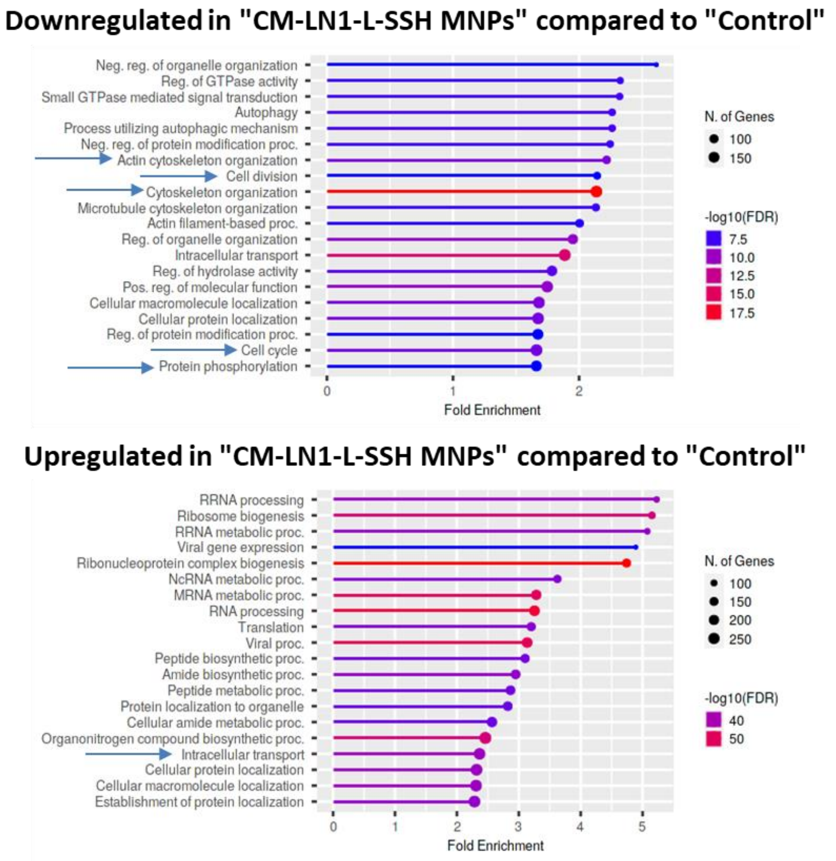

Figure S14. Proteomic analysis: complete list of the GO terms related to the “CM-LN1-SSH MNPs +AMF” experimental class. Arrows indicate the regulated GO terms discussed in the main text.

MNP sample	d_{TEM} (nm)	a(Å)	d_{XRD} (nm)
**SH**	19.2±1.2	8.398(4)	17.6(3)
**SHS**	21.9±0.8	8.392(7)	19.2(1)
**SS**	20.6±0.5	8.415(9)	18.9(1)
**SSH**	23.0±0.8	8.409(2)	20.6(6)

Table S1 Structural parameters of studied samples (SH, SHS, SS, and SSH MNPs). d_{TEM}: the mean size of nanoparticles calculated from TEM micrographs; a: lattice parameter; d_{XRD}: the mean crystallite size of nanoparticles calculated from XRD patterns. Numbers in parentheses indicate the estimated standard deviation of the last significant digit calculated using Pawley refinement.

	ICP-OES				XPS			
**MNP sample**	**Co (%)**	**Fe (%)**	**Mn (%)**	**Zn (%)**	**Co (%)**	**Fe (%)**	**Mn %)**	**Zn (%)**
**SH**	8.7	86.0	0	5.2	18.9	68.8	0.0	12.2
**SHS**	2.2	93.6	3.3	0.9	2.3	82.1	14.6	1.0
**SS**	0	86.3	5.2	8.7	0.0	49.6	22.4	28.0
**SSH**	3.5	90.7	1.8	4.1	19.6	76.0	2.2	2.2

Table S2. Elemental composition of as-synthesized samples (SH, SHS, SS, and SSH MNPs) determined by ICP-OES and XPS.

MNP sample	M_S (Am^2/kg)	M_r (Am^2/kg)	H_c (kA/m)	SAR (W/g)	ILP (nHm^2/kg)
**SH**	65.7	14.4	50.9	38.5±0.6	1.56±0.02
**SHS**	68.2	11.7	33.4	61.3±1.6	2.49±0.05
**SS**	71.4	0.45	3.5	45.1±0.8	1.83±0.03
**SSH**	77.1	0.55	5.5	69.6±1.4	2.82±0.08

Table S3. Magnetic parameters of studied samples (SH, SHS, SS, and SSH MNPs). M_S: saturation magnetization; M_r: remanence magnetization; H_c: coercive field; *SAR*: specific absorption rate; *ILP*: intrinsic loss power. SAR and ILP results are expressed as mean ± standard deviation (SD).

	DLS		Z-potential	TGA	
**MNP sample**	**Size (nm)**	**PDI**	**Z-potential (mV)**	**Organic mass (%)**	**Inorganic mass (%)**
**L-SSH**	293±8	0.312±0.009	-12.4±0.611	6.7	93.3
**CM-L-SSH**	329±6	0.464±0.019	-13.9±0.119	7.7	92.3
**LN1-L-SSH**	304±6	0.396±0.007	-11.8±0.689	7.4	92.6
**CM-LN1-L-SSH**	334±12	0.554±0.042	-14.7±0.153	9.3	90.7

Table S4. DLS, Z-potential, and TGA results for L-SSH, CM-L-SSH, LN1-L-SSH, and CM-LN1-L-SSH MNPs. *PDI*: polidispersity index.
